# The bistable mitotic switch in fission yeast

**DOI:** 10.1091/mbc.E24-03-0142

**Published:** 2024-05-06

**Authors:** Béla Novák, John J. Tyson

**Affiliations:** a Department of Biochemistry, Oxford University, Oxford OX1 3QU, UK; b Department of Biological Sciences, Virginia Polytechnic Institute & State University, Blacksburg, VA 24061; New York University

## Abstract

In favorable conditions, eukaryotic cells proceed irreversibly through the cell division cycle (G1-S-G2-M) in order to produce two daughter cells with the same number and identity of chromosomes of their progenitor. The integrity of this process is maintained by “checkpoints” that hold a cell at particular transition points of the cycle until all requisite events are completed. The crucial functions of these checkpoints seem to depend on irreversible bistability of the underlying checkpoint control systems. Bistability of cell cycle transitions has been confirmed experimentally in frog egg extracts, budding yeast cells and mammalian cells. For fission yeast cells, a recent paper by Patterson *et al.* (2021) provides experimental evidence for an abrupt transition from G2 phase into mitosis, and we show that these data are consistent with a stochastic model of a bistable switch governing the G2/M checkpoint. Interestingly, our model suggests that their experimental data could also be explained by a reversible/sigmoidal switch, and stochastic simulations confirm this supposition. We propose a simple modification of their experimental protocol that could provide convincing evidence for (or against) bistability of the G2/M transition in fission yeast.

## Abstract (executive summary)

Based on their elegant experiments, Patterson *et al.* (2021) concluded that the G2/M transition in fission yeast is controlled by a bistable switch, but we question that conclusion.

Using detailed stochastic simulations, we show that the coexisting low and high Cdk1 activity states observed by Patterson et al. can be reproduced by either an irreversible/bistable or a reversible/ultrasensitive switch, and our analysis shows why the experiments of Patterson et al. are inconclusive.

We suggest a decisive experiment to distinguish unequivocally between an irreversible/bistable and a reversible/ultrasensitive switch.

## INTRODUCTION

Proliferating eukaryotic cells alternate between two phases: interphase and mitosis (M phase). Throughout interphase (G1-, S-, and G2 phases) chromosomes are decondensed. During S phase every chromosome is replicated to produce two identical sister chromatids held together by cohesin rings. In contrast, chromosomes are condensed during M phase, which prepares sister chromatids for segregation during anaphase (Morgan, 2007). Maintaining the ploidy of the cell requires that chromosome-replicating interphase strictly alternates with chromatid-segregating M phase. This requirement is satisfied by the irreversibility of the transitions between interphase and M phase (at the G2/M boundary) and mitotic exit (anaphase-telophase-cell division). In 1993, we proposed that irreversibility of these transitions is a consequence of bistability in the molecular mechanism controlling entry into and exit from mitosis (Novak and Tyson, 1993a,b). This mechanism is based on cyclin activation of mitosis promoting factor (MPF). Accumulation of “mitotic” cyclin in interphase triggers abrupt activation of MPF and entry into mitosis (Solomon *et al.*, 1990). Degradation of mitotic cyclin in anaphase and telophase leads to an abrupt loss of MPF activity as the cell divides and enters G1 phase. “Bistability” of the mitotic switch means that the cyclin threshold for mitotic entry is larger than the threshold for mitotic exit; for cyclin concentrations between these two thresholds, the M-phase control system can be either in an interphase state (low level of MPF activity) or in a mitotic state (high level of MPF activity). For an interphase cell to enter mitosis, the level of mitotic cyclin must increase above the “entry” threshold, and then, to return to interphase, mitotic cyclin must be degraded to a level below the “exit” threshold. We proposed that this behavior, known as hysteresis, is the basis of the alternation between interphase and mitosis during cell proliferation (Novak and Tyson, 1993b).

Based on the evolutionary conservation of the mitotic control network (Nurse, 1990), we applied this concept of a bistable mitotic switch to both the free-running cell cycles of early Xenopus embryos (nongrowing cells) and to the growth-controlled cell cycles of fission yeast (Novak and Tyson, 1993a,b, 1995). Dedicated experiments with Xenopus cell-free extracts confirmed these predictions by showing that more mitotic cyclin is required for mitotic entry than for maintaining the extract in M phase (Pomerening *et al.*, 2003; Sha *et al.*, 2003). Later, we applied the bistability concept to the G1/S transition in budding yeast and mammalian cells (Tyson *et al.*, 1995; Chen *et al.*, 2000; Novak and Tyson, 2004). The distinctive signatures of bistability were observed experimentally in budding yeast (Cross *et al.*, 2002; Lopez-Aviles *et al.*, 2009) and in a human (HeLa) cell line (Yao *et al.*, 2008; Rata *et al.*, 2018), suggesting that bistability is a conserved feature of the cell-cycle transitions in eukaryotes.

Interestingly, experimental evidence for bistability of mitotic control in fission yeast has been missing. In 2021 Nurse’s group, using quantitative fluorescence microscopy, measured cyclin-CDK activity (i.e., MPF) as a function of total cyclin-CDK level in fission yeast cells and observed that cells close to the G2/M transition (cell length ≈ 12 μm) and containing equivalent amounts of cyclin-CDK could be either in G2 phase (low cyclin-CDK activity) or in M phase (high cyclin-CDK activity), which they interpreted as evidence that the dose-response curve is “clearly bistable, with cells existing in either an ‘on’ or an ‘off’ state (Patterson *et al.*, 2021)”. Intrigued by these results, we investigate their experimental observations in this study with a model of bistability in the activation of cyclin-CDK in fission yeast. Using a reliable stochastic simulation algorithm (SSA) (Gillespie, 2007), we show that our model yields dose-response curves in near-perfect agreement with Patterson *et al.*’s (2021) observations of “wildtype” cells and three mutant strains with aberrant activation of cyclin-CDK. Our analysis of these simulations and the experimental data shows, furthermore, that Patterson’s protocol probes only the cyclin threshold for mitotic entry (i.e., CDK activation) and not the cyclin threshold for mitotic exit (CDK inactivation). Therefore, the experiments do not provide unequivocal evidence for bistability. Indeed, we show that Patterson *et al.*’s (2021) experimental data are also consistent with a reversible (ultrasensitive, not bistable) mitotic transition, and we propose a modified experimental protocol that could provide unambiguous evidence for bistability in the mitotic control system of fission yeast.

### The experiment

To study the dependence of mitotic entry on the accumulation of mitotic CDK activity in fission yeast, Patterson *et al.* (2021) created a strain carrying a temperature-sensitive Cdk1 mutation (*cdc2^ts^*) and a tetracycline-inducible, GFP-tagged Cdc13^dbΔ^-Cdk1 fusion protein (*TetP*:*cdc13^dbΔ^*-*GFP*-*cdc2*). The cyclin component of the fusion protein lacked the destruction box of the wildtype *cdc13^+^* gene; hence, their fusion protein is not degraded after the induced cell enters mitosis. Cells were also equipped with a fluorescently-tagged Cut3-based CDK activity sensor (*synCut3-mCherry*), which accumulates in the nucleus in its CDK-phosphorylated form. In their experiments, Patterson *et al.* (2021) first raised cells to the restrictive temperature to inactivate the cell’s endogenous supply of wildtype Cdk1 and then induced synthesis of the Cdc13^dbΔ^-Cdk1 fusion protein (denoted C-CDK, with the understanding that the cyclin component is non-degradable; a distinction that will become important later). Using single-cell assays, they simultaneously measured C-CDK level (arbitrary units of GFP fluorescence), CDK activity (arbitrary units mCherry nuclear fluorescence) and cell size (length in μm) at increasing times after induction of the fusion protein. The level of induced C-CDK in a cell depended on the length of induction rather than cell size (as in normal cell-cycle progression). In this way Patterson *et al.* (2021) managed to characterize the dependence of the mitotic cyclin threshold (i.e., fusion protein level) for CDK activation on cell size. As a check on the underlying mechanism of the mitotic control system, they also studied a form of the fusion protein, Cdc13^dbΔ^-Cdk1^AF^ (denoted C-CDK^AF^), that cannot be inactivated by inhibitory phosphorylation of the Cdk1 subunit by Wee1/Mik1 kinase. In addition, they did experiments in a *ppa2Δ* genetic background, where one of the type-2A protein phosphatases (PP2A) has been deleted. In their “wildtype” strain (i.e., *cdc13^dbΔ^-cdc2^+^ppa2^+^*), the authors observed a wide range of fusion protein levels where cells with both low and high CDK activity coexist.

Patterson *et al.* (2021) interpreted their observations as in vivo confirmation of the bistable “C-CDK activity vs C-CDK level dose-response curves previously demonstrated in vitro” by Sha *et al.* (2003) and Pomerening *et al.* (2003) in frog egg extracts. Although, we would like to believe that the mitotic control system in fission yeast is (as we predicted) governed by a bistable switch, as claimed by Patterson *et al.* (2021), we will show here that their data corresponds more closely to an early study in frog egg extracts by Solomon *et al.* (1990), who observed a sigmoidal dependence of C-CDK (“MPF”) activity on C-CDK level (“total cyclin”). Neither the experiments of Solomon *et al.* (1990) in vitro nor those of Patterson *et al.* (2021) in vivo provide proof of “bistability” because these experiments probe only one half of the dose-response curve of a bistable switch (turning on the switch by raising the cyclin level). The experiments of Sha *et al.* (2003) and Pomerening *et al.* (2003), based on our earlier computational studies (Novak and Tyson, 1993b), provided convincing evidence of bistability of the mitotic switch by raising and lowering the cyclin level in frog egg extracts and showing that, at an intermediate level of cyclin, the extract could remain in a stable steady state of low or high MPF activity depending on whether the extract began in a state of low or high MPF activity (a phenomenon called “hysteresis”). By using stochastic and deterministic modelling of the fission-yeast mitotic switch, we show that the observations of Patterson *et al.* (2021) are consistent with both an irreversible/bistable mitotic switch and a reversible/sigmoidal mitotic switch. Although we suspect that the mitotic switch is bistable, a sigmoidal switch is not excluded by the experiments. To resolve this uncertainty, we propose a simple modification to Patterson’s protocol that would allow the C-CDK level to both rise and fall and thereby provide unequivocal evidence for or against a bistable mitotic switch.

## RESULTS AND DISCUSSION

### The model

**FIGURE 1:**
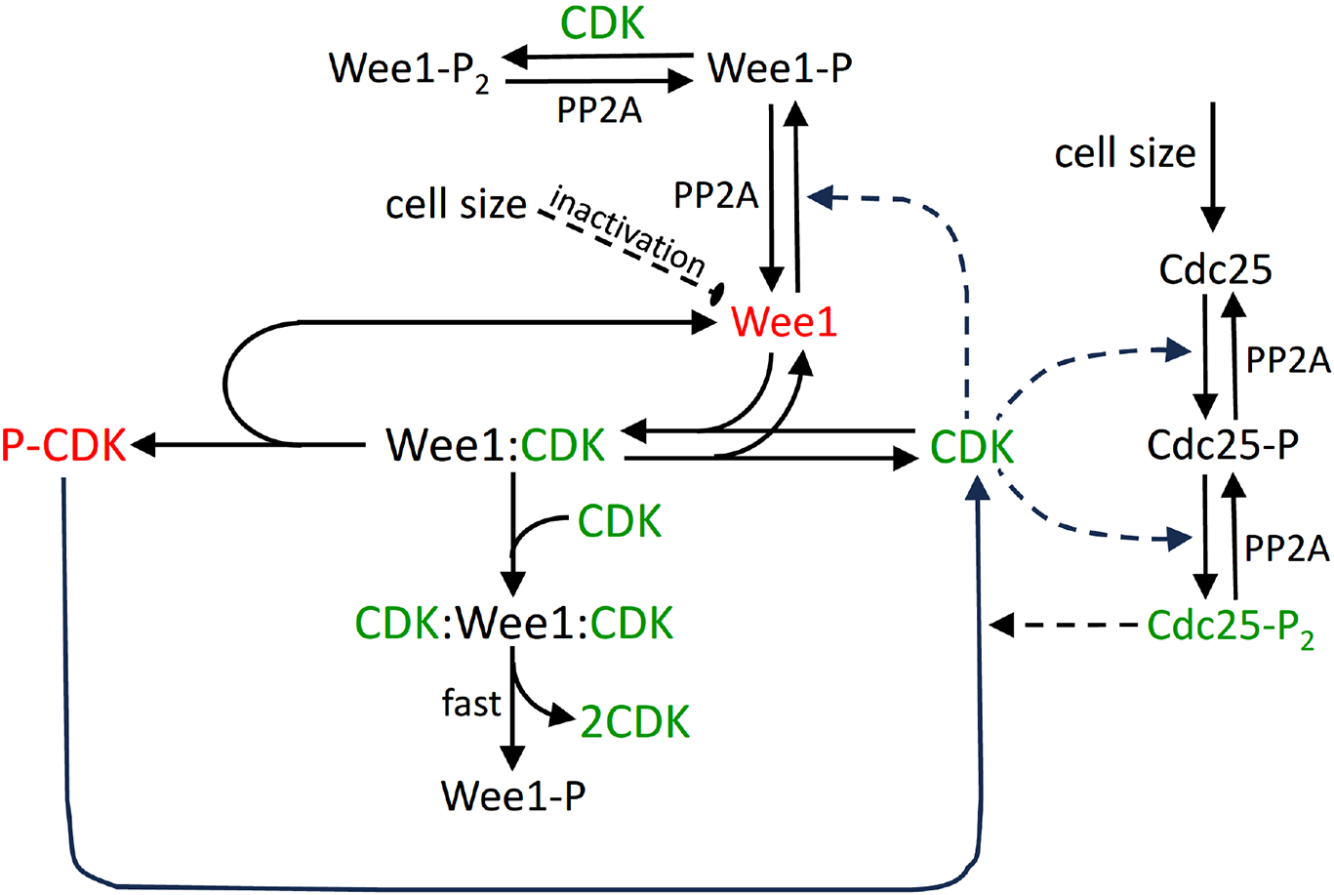
The molecular regulatory network of the model. Wee1 (red) represents the active forms of both Wee1 and Mik1 kinases. Active CDK (green) is inhibited by phosphorylation by Wee1 and reactivated by Cdc25 phosphatase (the active form is green). Wee1 forms a stoichiometric complex with CDK before catalyzing its inhibitory phosphorylation. The Wee1:CDK dimer can be attacked by active CDK to phosphorylate Wee1 and release two CDKs. Both Wee1 and Cdc25 are doubly phosphorylated by CDK, and the phosphorylations are reversed by PP2A. Because the active form of Wee1 is unphosphorylated and the active form of Cdc25 is doubly phosphorylated, the net activation of CDK is governed by two interlocked, positive feedback loops. Total Cdc25 concentration increases as the cell grows. Cell growth inhibits “Wee1” because the synthesis of Mik1 is restricted to small cells early in the cell cycle.

Our model of the mitotic control system in fission yeast is based on our previous work (Novak *et al.*, 1998; Tyson *et al.*, 2002; Gerard *et al.*, 2015; Chica *et al.*, 2016). C-CDK is phosphorylated on inhibitory sites of Cdk1 by Wee1/Mik1 kinases, and these phosphorylations are reversed by active Cdc25-phosphatase (Figure 1). Both the kinases and the phosphatase are phosphorylated by active C-CDK, but with opposite effects: Wee1 phosphorylations are inhibitory, Cdc25 phosphorylations are activatory. These positive feedback loops (− − and + +) are partially responsible for bistability of the mitotic switch. C-CDK phosphorylations of Cdc25 and Wee1/Mik1 are reversed by dephosphorylation by PP2A (Chica *et al.*, 2016). In our dynamic description of Wee1/Mik1-catalyzed phosphorylation of C-CDK we explicitly include the complex, Wee1:CDK, formed by the inhibitory kinases and their substrate (see Figure 1). This novel feature of our model provides additional positive feedback (− −) to make the mitotic switch bistable, and it contributes to weak bistability observed in the C-CDK^AF^ strain because Wee1 and Mik1 function as stoichiometric inhibitors of C-CDK even when the Cdk1 subunit cannot be phosphorylated. Stoichiometric inhibition of Cdk1 by Wee1/Mik1 is further supported by the observation that *mik1Δ wee1^ts^* double mutant cells at the restrictive temperature are less viable than *cdk1^AF^* cells (Gould and Nurse, 1989; Lundgren *et al.*, 1991). Consistent with experimental observations, our model has Cdc25 concentration increasing with cell size (Keifenheim *et al.*, 2017; Curran *et al.*, 2022). Also, the level of Wee1/Mik1 is cell-cycle regulated, for the following reason: although Wee1 is maintained at constant level, Mik1 protein level peaks early in the cycle due to induction by a cell cycle-regulated transcription factor, MBF (Christensen *et al.*, 2000; Ng *et al.*, 2001).

The model was also supplemented with a variable that accounts for the CDK activity sensor. Guided by experimental data, we assume that the unphosphorylated sensor rapidly equilibrates between cytoplasm and nucleus, but nuclear export of the phosphorylated form is attenuated (see *Materials and Methods*).

### Stochastic simulations

We simulated the temporal dynamics of the network in Figure 1 with Gillespie’s Stochastic Simulation Algorithm (SSA; Gillespie, 2007). Because our model of molecular interactions is formulated in terms of mass-action rate laws, Gillespie’s SSA provides reliable simulations of the stochastic fluctuation of the biochemical control system. Because the concentrations in our model are expressed in arbitrary units, we must adjust the volume of a typical newborn cell, V_0_ (also in AU), to provide a degree of stochastic fluctuations comparable to the “noise” observed in the experiments of Patterson *et al.* (2021) To this end, we chose V_0_ = 500. (Because a newborn fission yeast cell of length 7 μm and diameter 3 μm has a volume of 50 μm^3^ = 50 fL, our unit volume = 0.1 fL.) Simulations were run with different initial cell volumes for different lengths of time, allowing synthesis of fusion protein (C-CDK) during exponential volume growth. In the model, the rate of synthesis of C-CDK molecules is directly proportional to cell volume, and the molecules have a long half-life, because the fusion protein has a deleted destruction box. C-CDK concentration (#molec/volume) increases with time because the number of molecules of C-CDK is zero at the start of a simulation. At each time point we collected data for cell volume, V(t), C-CDK concentration, i.e., #C-CDK molecules/V(t), and CDK activity, i.e., nucleocytoplasmic ratio of the CDK-activity sensor. In this way stochastic simulations of the model provide predictions of how CDK activity depends on cell size and fusion protein concentration in single cells, as observed in the experiments. Each genetic background was analyzed by more than 6000 simulations.

**FIGURE 2:**
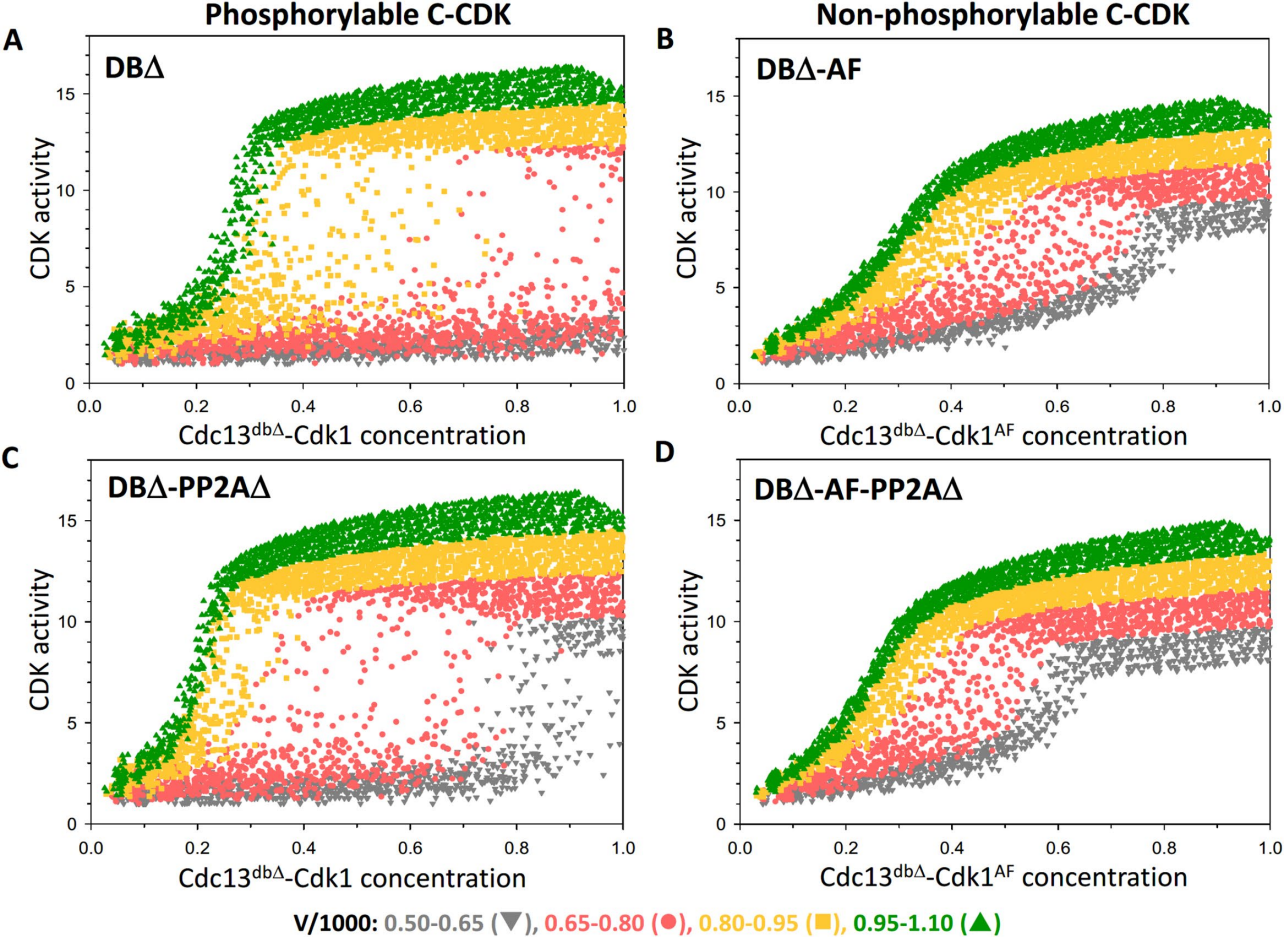
Stochastic simulations of the bistable mitotic-switch model. CDK activity is plotted as a function of fusion-protein concentration (#molec/V, V in arbitrary units) after induction of Cdc13^dbΔ^-Cdk1 (left column: A and C) and Cdc13^dbΔ^-Cdk1^AF^ (right column: B and D) in *pp2a*^+^ (top row: A and B) and *pp2aΔ*-deleted background (bottom row: C and D). Cells are sorted into four cell size bins (V = 1000 corresponds roughly to a dividing wildtype cell of length 14 μm and volume 100 fL).

In Figure 2 we plot CDK activity as a function of C-CDK concentration (AU), so that our figure is directly comparable with the original experimental data in Figure 2i of Patterson *et al.* (2021). We have grouped the cells into four cell-size bins (50–65 fL, 65–80 fL, 80–95 fL, and 95–110 fL, where 100 fL is the average size of a wildtype cell at division) similar to how Patterson *et al.* (2021) presented their experimental results in cell-length bins (7–9 μm, 9–11 μm, 11–13 μm, and 13–15 μm). Our simulations of phosphorylable and nonpho­sphorylable C-CDKs are provided on the left and the right panels of Figure 2, whereas the top and the bottom panels show the cases of *pp2a^+^* and PP2A-deleted cells. In Supplemental Figure S1, we plot the same simulations in terms of the concentration of the active form of C-CDK (i.e., number of unphosphorylated molecules per unit volume, in the same AU). While the CDK activity measured by the sensor saturates at high fusion-protein levels, consistent with experimental data, the concentration of active CDK increases linearly in the same region.

In Figure 2 and Supplemental Figure S1, three different types of cells (low, intermediate or high CDK activity) can be distinguished in each situation. Cells with low and high CDK activities are, respectively, before and after the process of CDK activation. At very small and very high C-CDK levels, all cells have, respectively, very low and very high CDK activity regardless of cell size. Cells with intermediate CDK activities are in the process of activating CDK, because no cell inactivates its CDK during simulations (or in the experiments). In both genetic backgrounds (*pp2a^+^* and *ppa2Δ*) and with both forms of fusion protein (phosphorylable and nonphosphorylable), CDK becomes activated at lower fusion protein levels in large cells (green) than in smaller cells (yellow and red; Figure 2; Supplemental Figure S1), clearly indicating that the cyclin (fusion protein) threshold for CDK activation is cell-size dependent. The lack of inhibitory CDK phosphorylation in the AF strain (CDK^AF^) reduces the cyclin threshold for CDK activation more than deletion of PP2A (Figure 2, B and C). The reduction of the size thresholds by CDK^AF^ and PP2AΔ are additive (the AF-PP2AΔ strain in Figure 2D).

### Deterministic model

**FIGURE 3:**
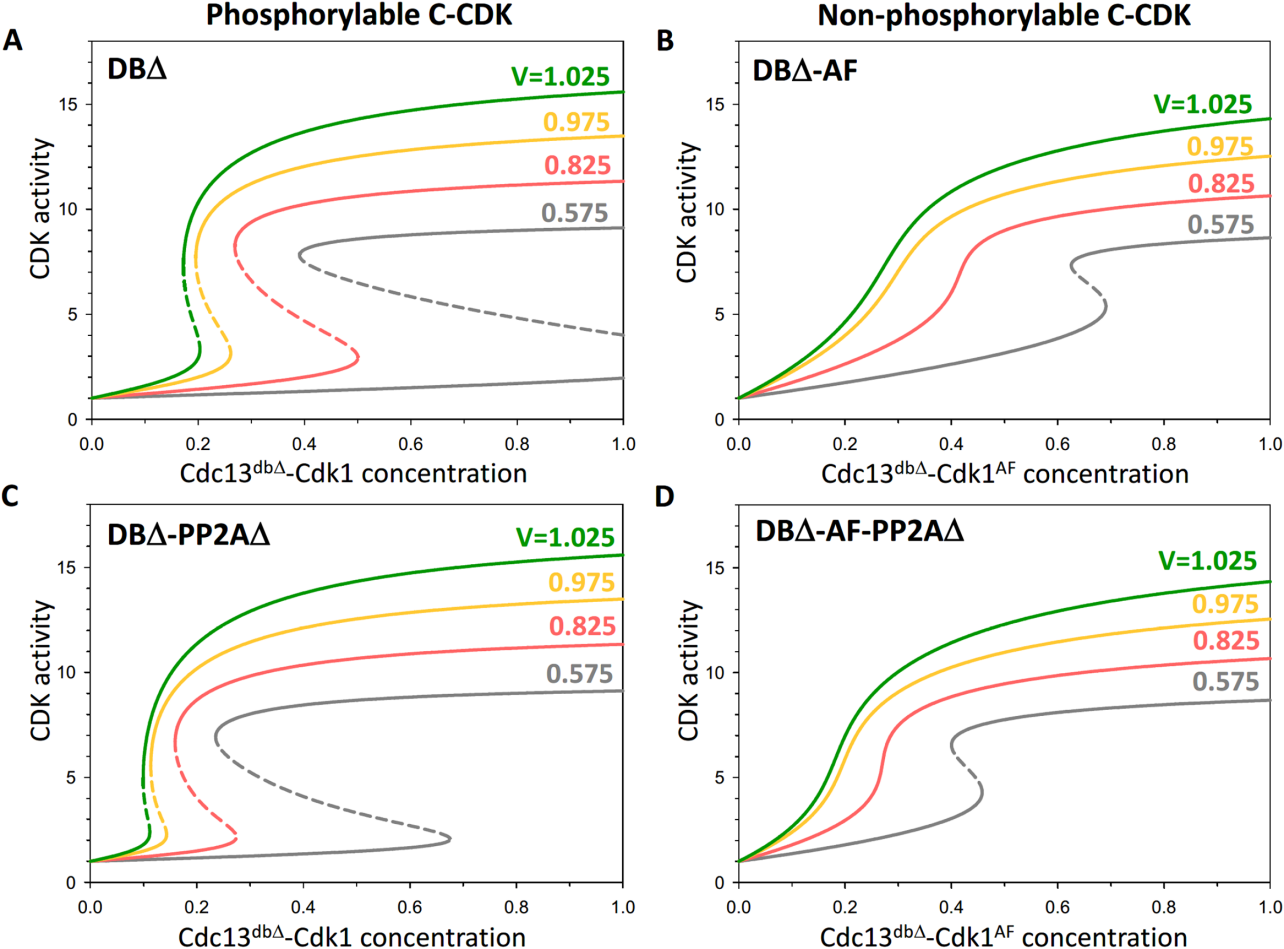
Dose-response curves for the dependence of the CDK-activity sensor on fusion-protein concentration predicted by the deterministic model. CDK-activity sensor is plotted as a function of fusion-protein concentration after induction of Cdc13^dbΔ^-Cdk1 (left column: A and C) and Cdc13^dbΔ^-Cdk1^AF^ (right column: B and D) in *pp2a*^+^ (top row: A and B) and *pp2aΔ*-deleted background (bottom row: C and D). CDK activities are plotted for fixed values of the parameter V in the deterministic model that corresponds to the middle of the cell-size bins defined for the stochastic simulations.

To gain some insight into the stochastic simulations in Figure 2, we analyze the corresponding deterministic model by means of one-parameter bifurcation diagrams (Figure 3) for each of the four strains (DBΔ, DBΔ-AF, DBΔ-PP2AΔ, and DBΔ-AF-PP2AΔ). Each bifurcation diagram depicts the dependence of the steady state value of the CDK activity sensor on the total fusion protein concentration in a cell of fixed volume (V = 1 corresponds to a dividing cell, 100 fL). The variables are plotted in the same AU used in Figure 2, for the stochastic model. (The corresponding bifurcation diagrams for the concentration of the active form of C-CDK are provided in Supplemental Figure S2.) For each strain, the dependence of CDK activity on fusion protein concentration is calculated for four cell volumes, corresponding to the middle values of the cell-size bins (57.5 fL, 72.5 fL, 87.5 fL, and 102.5 fL) in the stochastic simulations.

In the phosphorylable C-CDK strains, CDK activity versus fusion protein concentration is S-shaped at all four sizes; whereas, for the nonphosphorylable C-CDK strains, the bifurcation curves are S-shaped only for the smallest cells. The lower and the upper branches of the S-shaped curves are stable steady states corresponding to interphase and M-phase activity of CDK, respectively. In the phosphorylable C-CDK strains, the interphase and mitotic states are overlapping at intermediate concentrations of fusion protein, suggesting bistability in the mitotic control system. The low and high CDK activity states are connected by a middle branch of unstable steady states where the mitotic control system cannot stay for long in a stochastic environment. The termination of the lower steady state corresponds to the cyclin (fusion protein) threshold for CDK activation; this threshold decreases dramatically as a function of cell volume for the nonphosphorylable C-CDK strains. At the other end of the bistable range, the termination of the high CDK-activity state represents the mitotic cyclin threshold for CDK inactivation. In summary, at the heart of our model for the fission yeast mitotic control is a strong bistable switch with two different fusion-protein thresholds between the interphase and M-phase transitions. Small cells of the strains carrying the Cdk1^AF^ allele exhibit weak bistability in the absence of inhibitory CDK phosphorylation because of the mutual antagonism remaining between Cdk1^AF^ and Wee1; namely, unphosphorylated Wee1 binds to and inhibits Cdk1^AF^, and Cdk1^AF^ phosphorylates and inactivates Wee1.

The predicted range of bistability is dependent on cell size as well as genetic background and biochemical details. This dependence is best illustrated on a two-parameter bifurcation diagram, which depicts the boundaries between interphase and mitosis as functions of fusion protein concentration and cell volume (Supplemental Figure S3). High fusion-protein concentration and large cell size favor the mitotic state, while low fusion protein concentration and small cell size keep cells in interphase. The cyclin (fusion protein) thresholds for CDK activation and inactivation (the green and the red curves, respectively, in Supplemental Figure S3) correspond to the boundaries of interphase and mitotic states, and both thresholds are decreasing functions of cell size. As cell size increases, the two curves meet at a “cusp” point, which represents a merger of interphase and mitotic states at large cell size. The range of bistability can be estimated by the horizontal difference between the two curves, as illustrated by the dotted line at V = 0.6. Supplemental Figure S3B shows that, in the absence of inhibitory CDK phosphorylation (Cdk1^AF^), bistability is exhibited only by small cells (V<0.7) and only over a narrow range of C-CDK levels. Notice that the absence of PP2A shifts bistability to smaller C-CDK ranges (Supplemental Figure S3C), and the two effects (Cdk1^AF^ and PP2AΔ) are additive (Supplemental Figure S3D).

In the absence of inhibitory CDK phosphorylation, interphase and M-phase are not separated by a bistable switch with sharp thresholds, except for very small cells. Nonetheless, based on the data of Patterson *et al.* (2021), a boundary between interphase and M-phase can be defined by points where CDK activity = 5, which is indicated by the dashed lines in Supplemental Figure S3, B and D.

**FIGURE 4:**
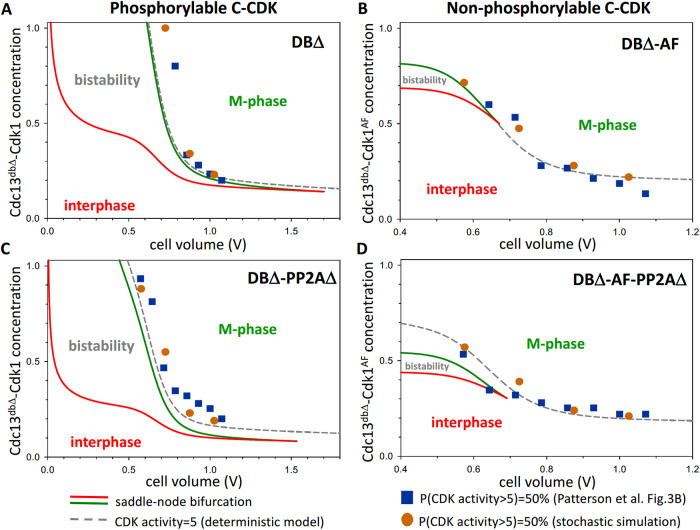
Cell-size dependence of fusion-protein thresholds for mitotic entry and exit in the deterministic model. Induction of Cdc13^dbΔ^-Cdk1 (left column: A and C) and Cdc13^dbΔ^-Cdk1^AF^ (right column: B and D) in *pp2a*^+^ (top row: A and B) and *pp2aΔ*-deleted background (bottom row: C and D). Solid lines depict the saddle-node bifurcation points for transitions into M phase (green line) and back to interphase (red line). Grey dashed lines depict the fusion protein concentration where CDK activity reaches the value of five. Orange circles and blue squares show fusion protein concentrations where more than 50% of cells have CDK activity higher than five in our stochastic simulations and in the experiments of Patterson *et al.* (2021), respectively.

To characterize their C-CDK activity data, Patterson *et al.* (2021) proposed an informative statistical measure: “the threshold C-CDK level required for 50% of cells to reach a C-CDK activity determined as being >5 in arbitrary units … within different size bins.” This measure is plotted as a function of cell length (μm) for their four different strains in Figure 3B of Patterson *et al.* (2021). Their figure is directly comparable to our model if we swap the axes of the two-parameter bifurcation diagram (Supplemental Figure S3 becomes Figure 4), and plot the C-CDK concentration required to cross CDK activity = 5 as a function of cell volume (the blue squares on Figure 4 are the experimental data points in Patterson’s Figure 3B). In the bistable regime (Figure 4, A and C), the experimental data lie slightly above the mitotic entry threshold of the bistable switch because of a short time-delay in CDK activation. When cell cycle progression is not controlled by bistability (nonphosphorylable C-CDK^AF^, Figure 4, B and D), the experimental points are spread around the CDK activity = 5 line calculated by the deterministic model. For our stochastic simulations, we plot–for each of the four cell volume classes–the C-CDK concentration where 50% of cells are above and 50% below CDK activity = 5, and these points (the orange circles on Figure 4) are in excellent agreement with the experimental data in all genetic backgrounds. We conclude that our bistable model provides a good quantitative fit of the experimental results reported by Patterson *et al.* (2021). But the agreement between experiments and modelling also clearly indicates that the experiments only scan the dependence of the mitotic entry threshold (the green curve) on cell size. That there is a lower threshold for mitotic exit (the red curve)–and hence a region of bistability–is not probed by the experimental protocol of Patterson *et al.* (2021).

### Dynamics of CDK activation in Patterson *et al.*’s (2021) experiments

**FIGURE 5:**
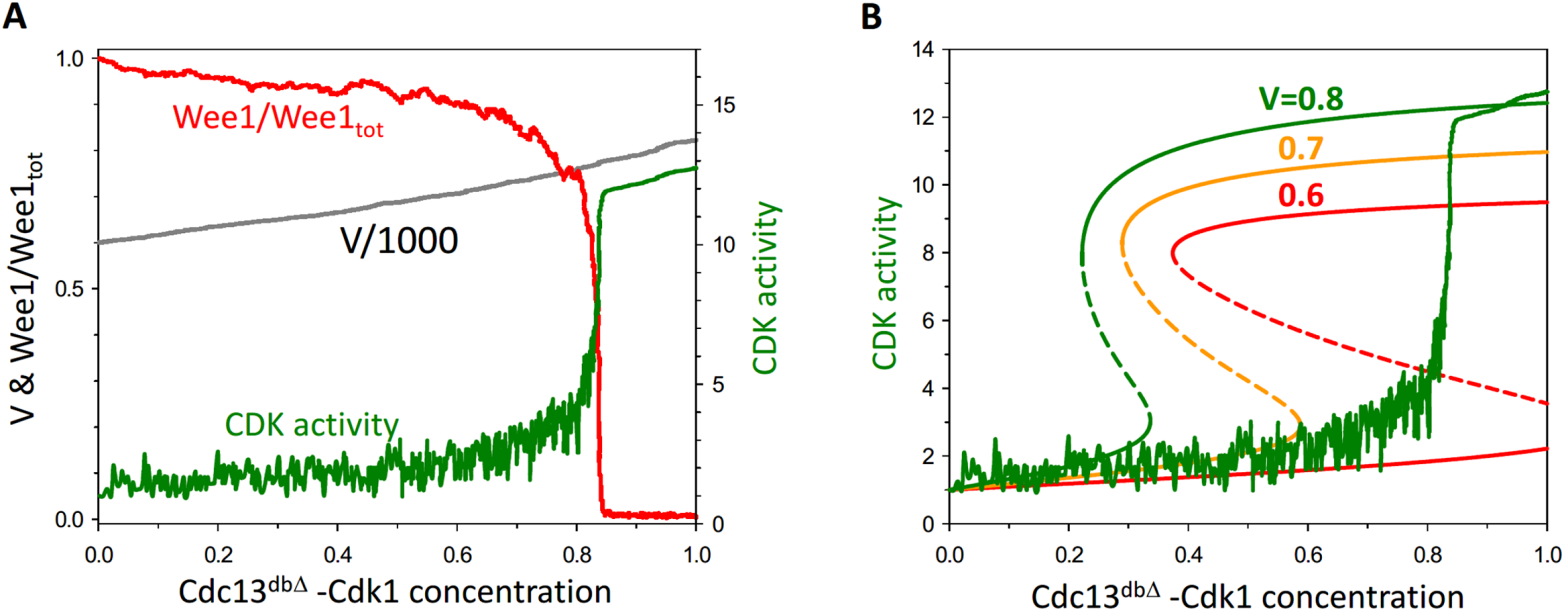
Comparison of the stochastic and deterministic models. (A) Stochastic simulation of mitotic entry induced by fusion protein in WT genetic background for a cell born with volume = 600. (B) Overlay of a stochastic simulation on a one-parameter bifurcation diagram comparable to Figure 3A.

To understand the dynamics of CDK activation in the experiments of Patterson *et al.* (2021), we analyzed our stochastic simulations (Figure 2) in the context of deterministic bifurcation curves (Figure 3). Figure 5A shows a representative stochastic simulation of CDK activation in a growing cell with V_0_ = 600 (i.e., 60 fL) at the start of fusion-protein induction. Because expression of fusion protein starts after inactivation of the endogenous Cdk1 molecules (*cdc2^ts^*), the CDK activity sensor equilibrates between cytoplasm and nucleus and shows an initial value of one (green curve, right axis). Wee1 is predominantly in its unphosphorylated, active form (Figure 5A), which keeps the cell on the lower (interphase) branch of the bifurcation diagram (Figure 5B), with CDK activity near one. Because fusion protein is being synthesized by the cell, the total C-CDK level moves to the right on both diagrams (Figure 5, A and B), and this is accompanied by an increase of cell volume, which causes a reduction in the cyclin (fusion protein) threshold for CDK activation (the S-shaped curves in Figure 5B are drifting to the left as the cell grows). When the fusion protein concentration reaches the right knee of the S-shaped curve, Wee1 becomes inactivated (Figure 5A) and CDK activity jumps to the upper (mitotic) branch of the bifurcation diagram. This analysis suggests that the experiments of Patterson *et al.* (2021) only probed one side of the bistable switch, namely the activation of CDK as the cell both grows and synthesizes additional fusion protein. Because larger cells activate their CDK at lower fusion-protein concentration than smaller cells, they observed coexisting low and high CDK activity states at a given fusion-protein concentration. The alternative steady states of the bistable mitotic switch do not play any role in creating overlapping low and high CDK activity states in Patterson *et al.*’s (2021) experiments. The only necessary requirements to observe overlapping low and high CDK activity states in these experiments are: (1) that the fusion-protein threshold for C-CDK activation be size-dependent, and (2) that the transition be abrupt. But the switch need not be bistable to reproduce the basic properties of Patterson *et al.*’s (2021) experiments.

### Stochastic simulation of a size-controlled reversible switch

To illustrate our argument that a nonbistable ultrasensitive mitotic switch is sufficient to create overlapping interphase and mitotic states, we simulated a model of a reversible switch. Bistability in our model is dependent on a positive feedback loop, whereby C-CDK inactivates Wee1, its inhibitory kinase and stoichiometric inhibitor. To convert our irreversible bistable switch into a reversible sigmoidal switch while maintaining the network structure in Figure 1, we assume that the phosphorylation of the Tyr-modifying enzymes (Wee1 and Cdc25) by C-CDK depends on fusion-protein concentration rather than CDK activity. Of course, this is a biochemically unrealistic assumption, but it serves to illustrate our point. Under this assumption, the bifurcation diagram (CDK activity vs. C-CDK level) of the mitotic control system becomes sigmoidal in all genetic backgrounds, for both CDK activity (Supplemental Figure S4) and for active CDK concentration (Supplemental Figure S5). Sigmoidal bifurcation diagrams are characteristic of a reversible, ultrasensitive switch. Observe that the threshold for CDK activation is still cell-size dependent (Supplemental Figures S4 and S5).

**FIGURE 6:**
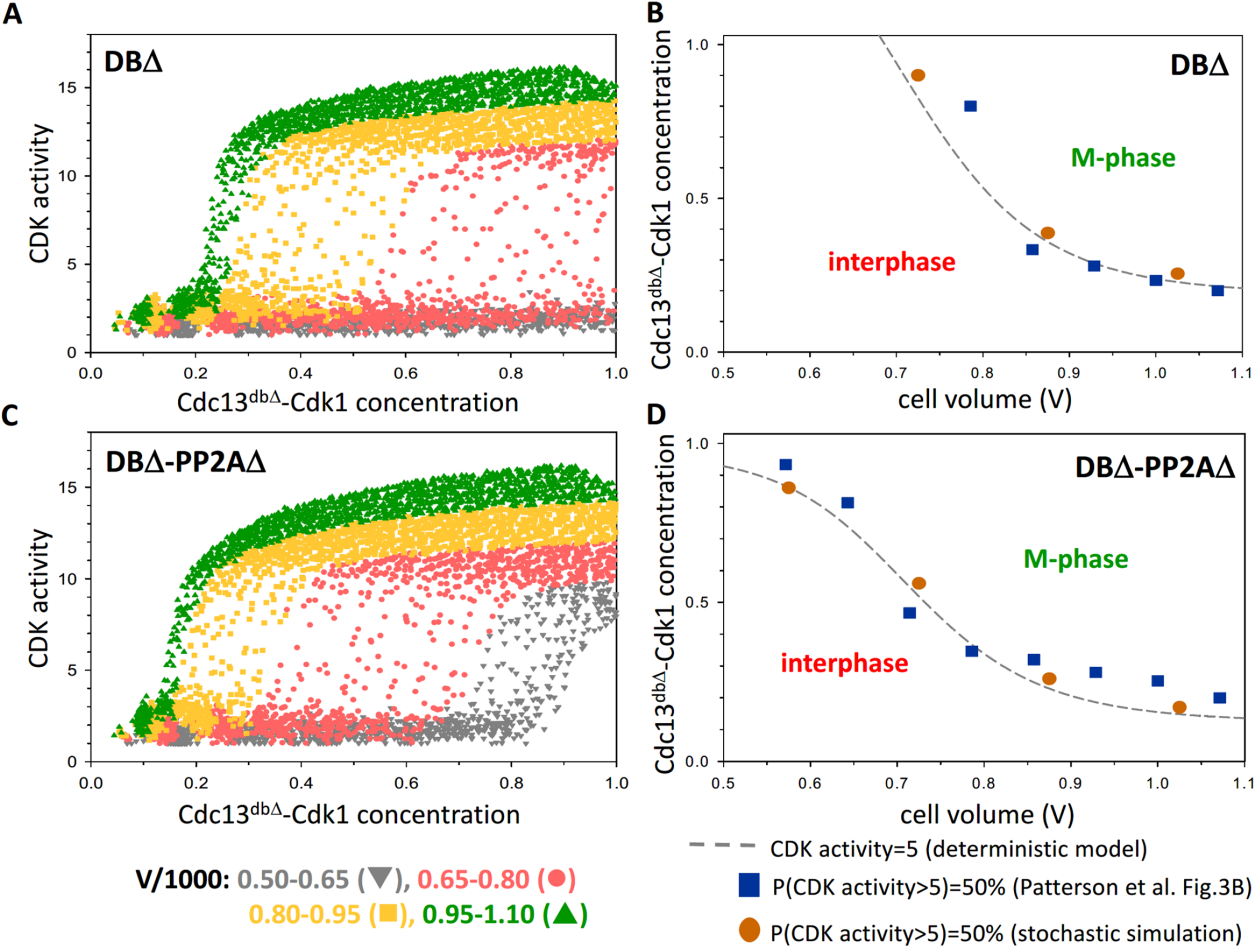
The reversible mitotic-switch model. (A and C) Stochastic simulations of Cdc13^dbΔ^-Cdk1 induction in *pp2a*^+^ and *pp2aΔ*-deleted backgrounds, respectively. (B and D) Cell size dependence of fusion-protein threshold for mitotic entry in *pp2a*^+^ and *pp2aΔ*-deleted backgrounds, respectively. The dashed grey lines depict where CDK activity = 5 in the deterministic model. The orange circles and blue squares indicate the fusion-protein concentrations where more than 50% of cells have CDK activity higher than five in our stochastic simulations and in Patterson *et al.* (2021) experiments, respectively.

Using this model, we ran stochastic simulations of the induction of nondegradable fusion protein and generated scatter plots of CDK activity versus C-CDK concentration, which were sorted into cell-size bins in the same manner as for the bistable model. Stochastic simulations of phosphorylable C-CDK with and without PP2A show wide ranges of overlapping low and high CDK activities at small size (Figure 6, A and C)–distributions that are indistinguishable from the bistable model. In addition, both deterministic and stochastic versions of the “reversible-switch model” (Figure 6, B and D) fit well with the data in Figure 3b of Patterson *et al.* (2021). (Stochastic simulations of C-CDK^AF^ with and without PP2A are shown in Supplemental Figure S6.) These simulations prove that a size-controlled, reversible (sigmoidal) switch is sufficient to create overlapping interphase and mitotic states at a fixed fusion-protein level; so the experiments of Patterson *et al.* (2021) do not unequivocally demonstrate that the C-CDK control system is bistable.

### Proof of bistability with degradable fusion protein

**FIGURE 7:**
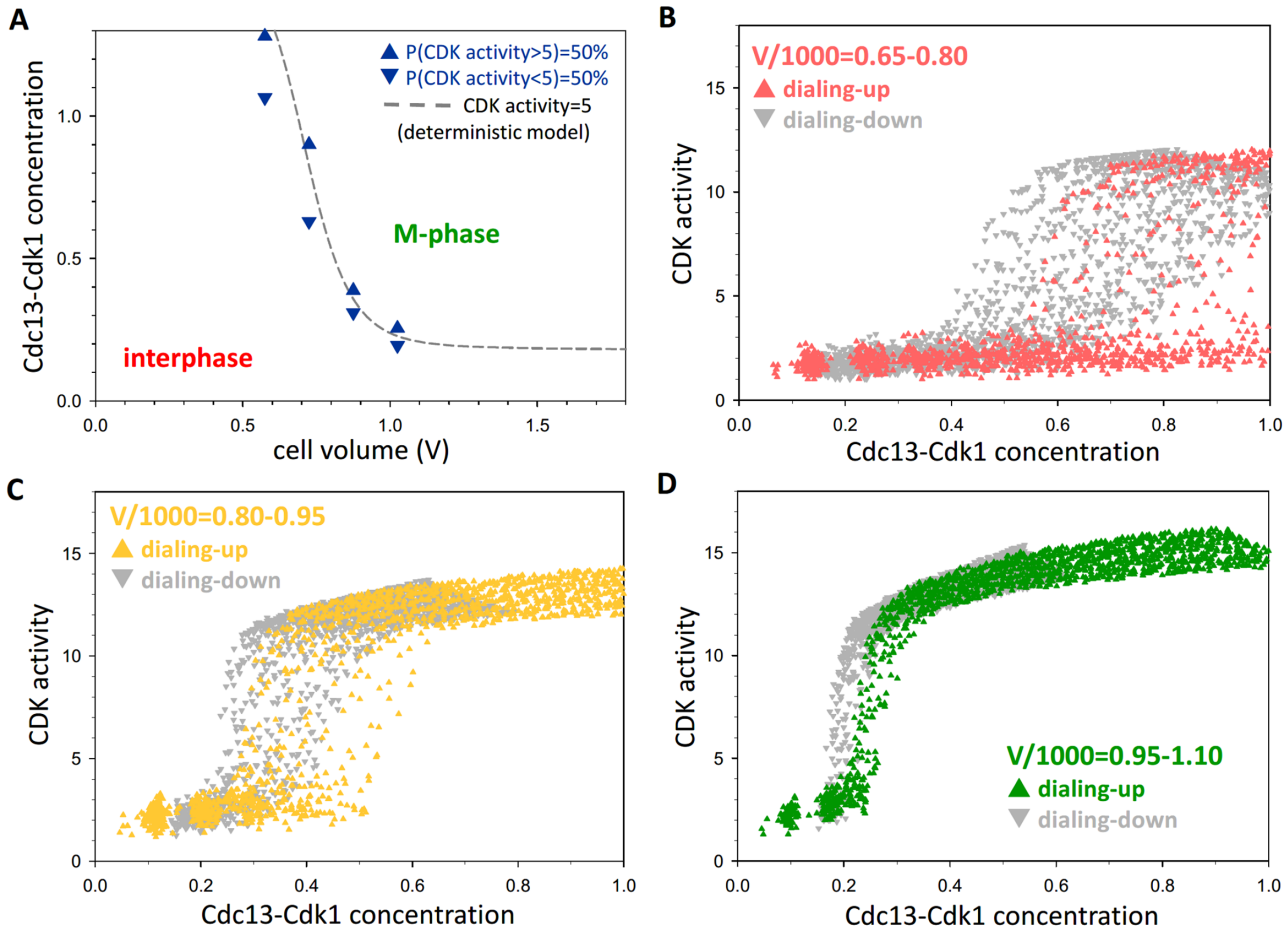
Reversibility of mitotic entry and exit in a monostable, ultrasensitive mitotic-switch model. (A) The dependence of fusion-protein thresholds on cell size. The dashed line depicts the fusion-protein concentration where CDK activity = 5 in our deterministic model. The triangles (▴ and ▾) indicate the fusion-protein concentrations where more than 50% of cells have CDK activity higher and lower than five, respectively, in our stochastic simulations. (B–D) Stochastic simulations of dialing-up the concentration of Cdc13^dbΔ^-Cdk1 (red, yellow and green data points) and dialing-down the concentration of degradable Cdc13-Cdk1 fusion-protein (grey data points). Cells are clustered according to size into small (panel B, V = 650–800), medium (panel C, V = 800–950) and large (panel D, V = 950–1100) size classes. During dialing-up, nondegradable C-CDK is induced for different times in cells that are initially in the low CDK activity state (interphase); during dialing-down, the level of degradable C-CDK is followed in cells that are initially in the high CDK activity state (M-phase) and are degrading the fusion protein.

If the mitotic transition were governed by a reversible switch whose thresholds for CDK activation and inactivation are identical, then the switch would activate and inactivate CDK at the same fusion-protein concentration regardless of whether its concentration were increasing or decreasing. To illustrate this point, we performed stochastic simulations of the reversible-switch model starting from the mitotic state (high CDK activity), assuming that the fusion protein is degradable (i.e., the WT strain in Table 1). The results of these stochastic simulations, indicated by the grey data points in Figure 7, B–D (overlaid on the previous simulations of mitotic entry induced by nondegradable fusion protein), show that cells inactivate CDK at a level of fusion protein that is only slightly smaller than the level where activation takes place (Figure 7A). This small offset in thresholds for CDK activation and inactivation is caused by time-delays in the activation and inactivation reactions.

**TABLE 1:** Fission yeast strains used in the experiments of Patterson *et al.* (2021) and discussed in this work.

Strain notation	Abbrev*	Genotype
Cdc13^dbΔ^-Cdk1^WT^	DBΔ	*cdc2^ts^ TetP:cdc13^dbΔ^-GFP-cdc2^+^ ppa2^+^ synCut3-mCherry*
Cdc13^dbΔ^-Cdk1^AF^	DBΔ-AF	*cdc2^ts^ TetP:cdc13^dbΔ^-GFP-cdc2^AF^ ppa2^+^ synCut3-mCherry*
Cdc13^dbΔ^-Cdk1^WT^	DBΔ-PP2AΔ	*cdc2^ts^ TetP:cdc13^dbΔ^-GFP-cdc2^+^ ppa2Δ synCut3-mCherry*
Cdc13^dbΔ^-Cdk1^AF^	DBΔ-AF-PP2AΔ	*cdc2^ts^ TetP:cdc13^dbΔ^-GFP-cdc2^AF^ ppa2Δ synCut3-mCherry*
Cdc13^WT^-Cdk1^WT^	WT	*cdc2^ts^ TetP:cdc13^+^-GFP-cdc2^+^ ppa2^+^ synCut3-mCherry*

*We use these abbreviations as shorthand for the four strains studied by Patterson *et al.* (2021) and a fifth strain (WT) proposed by us.

**FIGURE 8:**
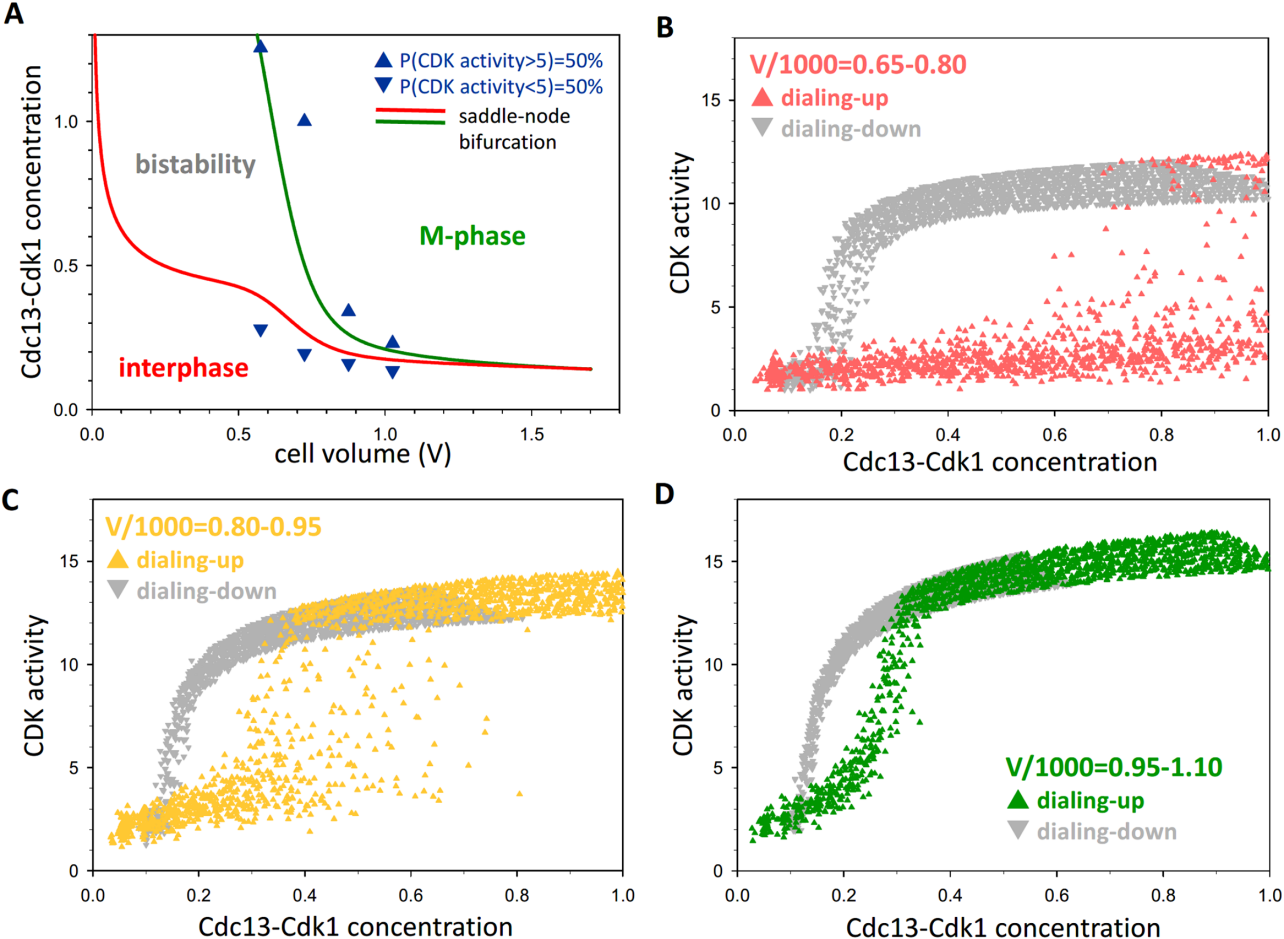
Hysteresis in the bistable mitotic-switch model. (A) The dependence of fusion-protein thresholds on cell size. The green and red lines are the saddle-node bifurcation points in our deterministic model where CDK activity abruptly rises and falls, respectively. The triangles (▴ and ▾) indicate fusion-protein concentrations where more than 50% of cells have CDK activity higher and lower than five, respectively, in our stochastic simulations. (B–D) Stochastic simulations of mitotic entry (dialing-up) and exit (dialing-down) with stable (Cdc13^dbΔ^-Cdk1) and unstable (Cdc13-Cdk1) fusion-protein, respectively. Cells are clustered according to their size into small (panel B, V = 650–800), medium (panel C, V = 800–950) and large (panel D, V = 950–1100) classes. During dialing-up, nondegradable C-CDK is induced for different times in cells that are initially in the low CDK activity state (interphase); during dialing-down, the concentration of degradable C-CDK is followed in cells that are initially in the high CDK activity state (M-phase) and are degrading the fusion protein.

A bistable mitotic switch is distinguished from a reversible switch by a significant difference in cyclin thresholds for CDK activation and inactivation. Repeating the stochastic simulations of the bistable mitotic-switch model with unstable fusion protein (WT strain) starting from the mitotic state (high CDK activity), we obtained the grey data points in Figure 8, B–D, overlaid on the previous simulations of mitotic entry induced by nondegradable fusion protein. (Note: we do not expect any significant difference in mitotic-entry dynamics between degradable and nondegradable fusion protein.) These panels illustrate the predicted outcome of the Patterson *et al.* (2021) experiments, if they were done with Cdk1^WT^ fused with degradable Cdc13^WT^. We draw attention to another prediction of the bistable model: the fusion-protein threshold for CDK inactivation is much less size-dependent than the threshold for CDK activation. As a consequence, the hysteresis effect is strongly cell-size dependent and most apparent for small cells which enter mitosis only at a high level of fusion protein and exit at a low level (Figure 8, A and B). Based on these results, we propose that a Patterson *et al.* (2021) protocol using fusion protein with an intact cyclin destruction-box could provide unequivocal evidence for bistability of the mitotic switch in fission yeast. Although cyclin degradation happens before cell division takes place, it might be useful to equip the cell with a thiamin-repressible *cdc11* gene (*nmt-cdc11*) and repress Cdc11 expression coincident with fusion-protein induction, which will block subsequent cell septation and separation.

## CONCLUSIONS

The eukaryotic cell division cycle proceeds, under favorable conditions, by a strict alternation of S phase (DNA replication) and mitosis (separation of the sister chromatids to opposite poles of the mitotic spindle). The proper sequence of these events is assured by “checkpoints” at three major “decision” points: the G1/S transition (commitment to DNA replication), the G2/M transition (entry into mitosis), and the metaphase/anaphase transition (exit from mitosis and back to G1). Each of these transitions is governed by a molecular mechanism that exhibits “bistable” dynamics (Novak and Tyson, 2021), that is, two stable steady states: a pretransition state (off) and a posttransition state (on). Checkpoint mechanisms maintain the bistable switch in the off state. Once the checkpoint is lifted and the cell passes the transition (flipping the switch on), it cannot (easily) revert to the off state, but rather, the cell returns to the pretransition state only by passing through all subsequent phases of the cell cycle. In this sense, progression through the eukaryotic cell cycle is a sequence of irreversible transitions governed by bistable switching mechanisms. This understanding of cell cycle dynamics, which is now widely accepted, has been established first by theoretical considerations (Novak and Tyson, 1993b; Thron, 1996, 1997; Chen *et al.*, 2000; Tyson *et al.*, 2002; Qu *et al.*, 2003a, b) and later by elegant and convincing experimental tests (Cross *et al.*, 2002; Pomerening *et al.*, 2003; Sha *et al.*, 2003; Lopez-Aviles *et al.*, 2009; Rata *et al.*, 2018). Crucial to all these tests of the theory is the idea that the experimental protocol must flip the switch in two different directions (off to on, and on to off) by perturbations that leave the cell in the same “external” conditions but the switch in either the on or off state. That is the basic signature of bistability: the coexistence of two stable steady states.

Among the experimental investigations of bistability in the cell cycle, a recent paper by Patterson *et al.* (2021) on the G2/M transition in fission yeast seems to confirm the coexistence of on and off states of cyclin-dependent kinase (CDK) activity in cells of similar sizes. Stochastic simulations of a reasonable model of CDK control in the mutant strains used by Patterson *et al.* (2021) quickly confirmed to us that a bistable control system would predict behavior much like that observed by Patterson *et al.* (2021). However, our simulations confirmed our suspicion that their experimental protocol (using nondegradable cyclin, Cdc13^dbΔ^) limited them to observe the off-to-on transition, only. Although they observed a broad range of cell sizes for which CDK activity could be either high or low, their results could be a consequence of stochastic fluctuations in a model of a reversible sigmoidal switch, provided the switching point is size dependent. We confirmed this suspicion by stochastic simulations of an alternative, sigmoidal switch without bistability. Thus, we conclude that the results of Patterson *et al.* (2021) do not provide convincing evidence of bistable regulation of the G2/M transition in fission yeast.

It is instructive, at this point, to compare the work of Patterson *et al.* (2021) to a study by Acar *et al.* (2005) of the galactose-signaling network of budding yeast. Combining elegant experiments with sophisticated modeling, Acar *et al.* (2005) provided convincing proof of bistability (“cellular memory”) in this nutritional control system. They measured P*_GAL1_-YFP* expression (the response) as a function of galactose concentration in the growth medium (the signal), analogous to Patterson’s measurements of CDK activity as a function of C-CDK concentration in fission yeast cells. In Acar’s experiments, the endogenous *GAL80* gene was replaced by P*_TET_-GAL80* in order to maintain Gal80 protein concentration at a constant value determined by doxycycline concentration in the growth medium. The fixed Gal80p concentration in Acar’s cells is analogous to cell volume in Patterson’s experiments. In Figure 3b of Acar’s paper, the team plotted the regions of monostable-off, monostable-on and bistable signaling in dependence on their two control parameters, external galactose concentration and intracellular Gal80p concentration, analogous to our Figure 4. Because Acar’s experiments explored both the off → on and on → off transitions, they could show that their observed thresholds (the red circles) correspond closely to both saddle-node bifurcation curves predicted by their model. On the other hand, Patterson’s experiments (as analyzed in our Figure 4) probe only the off → on transition.

Both transitions could be probed by Patterson-type experiments with a minor change of protocol. The experiments should be repeated with a degradable version of the cyclin component of their fusion-protein construct (i.e., *cdc13^+^-GFP-cdc2^+^*). Some cells will synthesize a large quantity of the fusion protein (as evidenced by GFP fluorescence) to activate CDK activity (off → on) and enter mitosis. These cells will then degrade the fusion protein, and its concentration will drop. The crucial observation will be: does CDK activity flip back to the off state at about the same level of GFP fluorescence as it flipped on, or must it drop to a much smaller level before CDK is inactivated? The former case would suggest a monostable, ultrasensitive switch; the latter case would be convincing evidence of bistability. Stochastic simulations with our bistable-switch model show, furthermore, that a large difference in off-to-on and on-to-off thresholds should be clearly evident only in small cells induced to enter mitosis. In addition, our model predicts that the off-to-on threshold decreases much more rapidly with cell size than the on-to-off threshold, so the hysteresis loop of the bistable switch shrinks as cells grow.

## MATERIALS AND METHODS

All mathematical equations and computational methods are described and explained in detail in the Supplemental Text. We also provide the code for the deterministic and stochastic models in the form of “.ode” files (see the Supplemental ODE Files) that allow the users to reproduce our Figures with the free available software XPP/AUTO (www.math.pitt.edu/∼bard/xpp/xpp.html).

## Supplementary Material



## Abbreviations used:

CDK: cyclin-dependent kinase
DBΔ: destruction box deleted
MPF: mitosis promoting factor
PP2A: protein phosphatase 2A
SSA: stochastic simulation algorithm
WT: wildtype
