# Supplementary material for: The bistable mitotic switch in fission yeast
Source: Mol Biol Cell. 2024 May 6;35(6):ar77. doi: 10.1091/mbc.E24-03-0142 (PMC11238088; doi:10.1091/mbc.E24-03-0142)
Supplement: Supplementary file 1 [file mbc-35-ar77-s001.pdf]

Supplemental Materials

Molecular Biology of the Cell

Novák and Tyson

Supplementary Text

Computational methods

Deterministic model

The time-rates of change of components were described by ordinary differential equations (ODE). The ODEs are composed of mass-action rate laws for the biochemical reactions, written in terms of the concentrations (in arbitrary units) of the interacting proteins (see Suppl Table S1). At constant concentration of fusion-protein ($CycB_{tot}$), the ODEs for different forms of CDKs are given by:

$$\frac{dCDK}{dt} = R_{cdc25} \cdot PCDK - k_{asswee1} \cdot CDK \cdot Wee1 + (k_{diswee1} + k_{iwee1} \cdot AF \cdot CDK + k_{dwee1}) \cdot CDKWee1$$

$$\frac{dPCDK}{dt} = k_{wee1} \cdot CDKWee1 - R_{cdc25} \cdot PCDK$$

$$CDKWee1 = CycB_{tot} - CDK - PCDK$$

In these equations, CDK and PCDK are the unphosphorylated and phosphorylated forms of the fusion protein. (Note: 'CDK' is the name of a protein, '*CDK*' is its concentration.) Wee1 forms a complex with CDK (called CDKWee1), which has two fates (see Fig.1): Wee1 phosphorylates CDK and releases PCDK, or a second molecule of CDK phosphorylates the Wee1 subunit, releasing the first CDK moiety + Wee1-P. A third property of the complex CDKWee1 is to function as a stoichiometric inhibitor of CDK activity. The catalytic rate constant, k_{wee1}, for the phosphorylation of CDK by Wee1 is set to zero when simulating the $Cdk1^{AF}$ mutant strains.

Concurrently, PCDK is dephosphorylated by Cdc25 at a rate dependent on the phosphorylation state of Cdc25:

$$R_{cdc25} = k'_{cdc25} \cdot Cdc25_{tot} + \left(k''_{cdc25} - k'_{cdc25}\right) \cdot Cdc25P_2$$

We assume that doubly-phosphorylated Cdc25 has a higher activity than the mono- and un-phosphorylated forms, $k_{25}'' > k_{25}'$.

The changing concentration of the tyrosine kinases, Wee1 and Mik1 (here treated as a single variable, Wee1) is described by:

$$\frac{dWee1_{tot}}{dt} = R_{swee1} - k_{dwee1} \cdot Wee1_{tot}\,, \qquad \text{where } R_{swee1} = k'_{swee1} + \frac{k_{swee1} \cdot J_{wee1}{}^{n}}{J_{wee1}{}^{n} + V^{n}}$$

The first term in R_{swee1} represents a constant rate of synthesis (concentration/time) of Wee1 (Keifenheim *et al.*, 2017; Curran *et al.*, 2022), and the second term represents a cell-cycle regulated rate of synthesis of Mik1, which is restricted to cells of small size (Christensen *et al.*, 2000; Ng *et al.*, 2001).

By contrast, total Cdc25 concentration increases with cell volume V(t) (Keifenheim *et al.*, 2017; Curran *et al.*, 2022), so we write:

$$\frac{dCdc25_{tot}}{dt} = k_{scdc25} \cdot V - k_{dcdc25} \cdot Cdc25_{tot}$$

The multisite phosphorylations of Wee1 and Cdc25 are described as distributive and ordered processes with two steps:

$$\frac{dWee1}{dt} = R_{swee1} - k_{asswee1} \cdot CDK \cdot Wee1 + (k_{diswee1} + k_{wee1}) \cdot CDKWee1 - (k_{iwee1} \cdot AF \cdot CDK + k_{dwee1}) \cdot Wee1 + k_{awee1} \cdot PP2A \cdot Wee1P$$

$$\frac{dWee1P_2}{dt} = k_{iwee1} \cdot AF \cdot CDK \cdot Wee1P - (k_{awee1} \cdot PP2A + k_{dwee1}) \cdot Wee1P_2$$

$$\frac{dCdc25}{dt} = k_{scdc25} \cdot V + k_{icdc25} \cdot PP2A \cdot Cdc25P - (k_{acdc25} \cdot AF \cdot CDK + k_{dcdc25}) \cdot Cdc25$$

$$\frac{dCdc25P_2}{dt} = k_{acdc25} \cdot AF \cdot CDK \cdot Cdc25P - (k_{icdc25} \cdot PP2A + k_{dcdc25}) \cdot Cdc25P_2$$

where the mono-phosphorylated forms are calculated by conservation relations:

$$Wee1P = Wee1_{tot} - CDKWee1 - Wee1 - Wee1P_2$$

$$Cdc25P = Cdc25_{tot} - Cdc25 - Cdc25P_2$$

The parameter *AF* defines the specific activity of the unphosphorylated, active fusion protein: *AF* = 1 for the native fusion protein, and *AF* = 0.5 for the AF-mutant, in line with experimental data (Rudner *et al.*, 2000).

Calculation of the nucleocytoplasmic ratio of the CDK sensor

To compare our model simulations with the data of Patterson et al. (2021), we propose an equation to compute the spatial location of the Cut3-based biosensor (Cut3-mCherry) used to estimate CDK activity in their experiments. Unphosphorylated Cut3 shuttles rapidly between nucleus and cytoplasm, but, when phosphorylated by CDK, Cut3P accumulates in the nucleus. Since CDK is largely nuclear, the sensor (S_c and S_n, for cytoplasmic and nuclear forms of unphosphorylated Cut3) becomes phosphorylated in the nucleus, which reduces its rate of export (k_{out}') from the nucleus. The slowly exported, phosphorylated sensor (S_p) is rapidly dephosphorylated in the cytoplasm. In steady state, the rate of import is equal to the rate of export:

$$V \cdot k_{in} \cdot S_c = V \cdot k_{out} \cdot S_n + k'_{out} \cdot S_p$$

Since $k_{in} = k_{out}$: $S_c = S_n + \frac{k'_{out}}{V \cdot k_{out}} \cdot S_p$

Assuming steady state for the phosphorylated form $k_{psensor} \cdot CDK \cdot S_n = k'_{out} \cdot S_p$, the total concentration of sensor in the nucleus is

$$S_n^{tot} = S_n \cdot \left(1 + \frac{k_{psensor} \cdot CDK}{k'_{out}}\right)$$

Combining the last two equations, the nucleocytoplasmic ratio of the sensor is calculated by:

$$\frac{S_n^{tot}}{S_c} = \frac{V \cdot k_{out}}{k'_{out}} \cdot \frac{k'_{out} + k_{psensor} \cdot CDK}{V \cdot k_{out} + k_{psensor} \cdot CDK}$$

The deterministic model was used to calculate one- and two-parameter bifurcation diagrams using the freely available software XPPAut (Ermentrout, 2002). The values of the parameters are provided in Suppl. Table S2. Rate constants (*k*'s) have a dimension of min^{-1}, while other parameters are dimensionless. In these calculations, V = cell volume is treated as a bifurcation parameter.

Stochastic model

For time-course simulations, we implemented Gillespie's Stochastic Simulation Algorithm (SSA) by converting the rates of elementary reactions into propensity functions (Gillespie, 2007). The model needs to be supplemented with the dynamics of fusion protein synthesis and degradation; in the deterministic context, we would have the following ODE for the rate of increase of CycB concentration:

$$\frac{dCycB}{dt} = k_{scycb} - k_{dcycb} \cdot CycB$$

Gillespie's SSA provides exact stochastic simulations for chemical processes governed by mass-action rate laws (reaction rate proportional to the product of the reacting species), as are the reaction rates in our model. Because we do not explicitly model mRNA levels, our protein synthesis terms, like k_{scycb}

above, underestimate the fluctuations in protein production caused by relatively large fluctuations in mRNA numbers (Pedraza and Paulsson, 2008; Shahrezaei and Swain, 2008). This neglect of mRNA fluctuations likely causes the model to underestimate fluctuations in protein numbers.

To convert from 'concentration units' in the deterministic model to 'molecule numbers' in the stochastic model, we multiply every concentration by volume, for example:

$$\#\text{CDK} = V \cdot [\text{CDK}]$$

To get a stochastic model with molecular noise comparable to that observed in Patterson et al.'s experiments, we assume that a newborn cell has a volume of ~500 'units'. Since a newborn fission yeast cell is ~50 μm^3, 1 'volume unit' is 0.1 μm^3. To check this assumption, we note that the total 'concentration' of C-CDK in large cells ($V \approx 1000$ AU) just entering mitosis is ≈ 0.4 'concentration unit' in the stochastic model (see Fig.2A). This concentration would correspond to #CDK ≈ 400 molecules, which in a cell of volume 100 fL would be a concentration of ~7 nM. This estimate is lower than we might expect for CDK concentrations in a mitotic yeast cell (50 nM). In addition to our neglect of mRNA fluctuations, we are also ignoring contributions of experimental noise to the data; so, it is not unreasonable that we must underestimate the numbers of fluctuating protein molecules in order to get a good fit to experimental observations.

In our stochastic simulations, we assume that cell volume is increasing exponentially, $V(t) = V_0 e^{\mu t}$. Although fission yeast cells actually grow in a piecewise linear fashion (Mitchison, 2003), the simpler exponential growth law (with doubling time ≅ 140 min) is perfectly adequate for our purposes in this paper. We take the average cell volume at birth V_0 = 500 AU = 50 fL. Stochastic simulations were started with initial cell volume of 400 (25% below the average birth size) followed by 25 AU increments until twice the average birth size (1000 AU) is reached. The rate of increase in numbers of C-CDK molecules is set proportional to the actual cell volume. The half-life of C-CDK was chosen ~6 h, because the fusion protein was made non-degradable by deleting its destruction box. Dilution of C-CDK molecules as the cell grows is taken into account by the dependence of reaction propensities on V(t). Simulations were designed to mimic the experimental protocol of Patterson et al. For each of 25 initial cell volumes, evenly distributed between 400 and 1000 AU, we initiated 251 'cells' with initially 0 molecules of fusion protein, and simulated the stochastic model, stopping the first cell at 4 molecules of C-CDK, the second at 8, the third at 12, … and the 251st at 1000 molecules of C-CDK. Each one of the 6275 simulations corresponds to one cell fixed and measured in the experimental protocol. For each simulated cell, we collected data for its volume, C-CDK concentration (number/volume) and CDK activity (nucleocytoplasmic ratio of the sensor) at the end of the simulation. We repeated the process of simulated data collection for each genetic background. In summary, stochastic simulations provided a scan of how CDK activity depends on cell size and fusion-protein concentrations in a manner comparable to the experimental data collection.

We provide the code for the Gillespie stochastic simulation algorithm (SSA) of our model as an 'ode' file for XPPAut. In each step of the SSA, the volume of the cell is increasing according to an exponential function, and, consequently, the propensities of the volume-dependent steps are, in principle, changing with time; and this time-dependence could be taken into account explicitly in implementing Gillespie's SSA (Shahrezaei *et al.*, 2008). However, the step-size between SSA updates is less than 1 s compared to the mass-doubling time (140 min) of cell growth. So, it is warranted to neglect the change in V(t) between steps of the SSA, as in our code.

Table S1. Dynamic variables of the deterministic and stochastic models of cell cycle control in fission yeast.

The protein's name refers to its concentration (AU) in the deterministic model or to its number of molecules per cell in the stochastic model. V is a parameter in the deterministic model (V=0.5 for a newborn cell of length 7 µm). V is an exponentially increasing function of time in the stochastic model (V=500 for a newborn cell of volume 50 µm^3).

CycB	Cdc13$^{db\Delta}$ -Cdk1 fusion protein; a parameter in the deterministic model
CDK	active (unphosphorylated) form of Cdc13$^{db\Delta}$ -Cdk1 or Cdc13$^{db\Delta}$-Cdk1^{AF}
pCDK	inactive (phosphorylated) form of Cdc13$^{db\Delta}$ -Cdk1
Wee1$_{tot}$	sum of all forms of Wee1/Mik1 inhibitory protein-kinases
Wee1	unphosphorylated, active form of Wee1/Mik1 inhibitory protein-kinases
Wee1P	mono-phosphorylated, inactive form of Wee1/Mik1 inhibitory protein-kinases
Wee1P$_2$	double-phosphorylated, inactive form of Wee1/Mik1 inhibitory protein-kinases
Cdc25$_{tot}$	sum of all forms of Cdc25 activatory protein-phosphatase
Cdc25	unphosphorylated, less active form of Cdc25 activatory protein-phosphatase
Cdc25P	mono-phosphorylated, less active form of Cdc25 activatory protein-phosphatase
Cdc25P$_2$	Double-phosphorylated, active form of Cdc25 activatory protein-phosphatase
sensor	CDK-activity sensor
V	cell volume

Table S2. Parameter values of the fission yeast cell cycle model.

Cdc13$^{db\Delta}$-Cdk1 synthesis/degradation:	$k_{scycb} = 0.02$,	$k_{dcycb} = 0.002$
Cdc13$^{db\Delta}$-Cdk1 phosphorylation by Wee1/Mik1:	$k_{asswee1} = 5$, $k_{wee1} = 1$	$k_{disswee1} = 1$,
Wee1/Mik1 synthesis & degradation:	$k'_{swee1} = 0.005$, $k_{dwee1} = 0.005^{ref}$, $n = 10$	$k_{swee1} = 0.02$, $J_{wee1} = 0.65$,
Cdc25 synthesis & degradation:	$k_{scdc25} = 0.05$,	$k_{dcdc25} = 0.05^{ref}$
Specific activity of Cdc25 forms:	$k'_{cdc25} = 0.1$,	$k''_{cdc25} = 5$
Wee1/Mik1 phosphorylation/dephosphorylation:	$k_{iwee1} = 10$,	$k_{awee1} = 1$
Cdc25 phosphorylation/dephosphorylation:	$k_{icdc25} = 1$,	$k_{acdc25} = 10$
CDK-activity sensor dynamics:	$k'_{out} = 0.06$, $k_{psensor} = 10$,	$k_{out} = 1$, $\varepsilon = 10$
Cdc13$^{db\Delta}$-Cdk1 activity:	AF=1 for Cdc13$^{db\Delta}$-Cdk1 and AF=0.5 for Cdc13$^{db\Delta}$-Cdk1^{AF}	
PP2A activity:	PP2A=1 for ppa2^{+} and PP2A=0.5 for ppa2Δ	
Newborn cell volume and growth rate:	V_0=500,	μ=0.005 min^{-1}

All rate constants (k's) have dimension of min^{-1}; all other parameters are dimensionless. Most of the parameters were tuned by hand to fit the data in Patterson et al. (2021), except for k_{dwee1} and k_{dcdc25}, which were estimated from data in (Keifenheim *et al.*, 2017).

Supplementary references:

Christensen, P.U., Bentley, N.J., Martinho, R.G., Nielsen, O., and Carr, A.M. (2000). Mik1 levels accumulate in S phase and may mediate an intrinsic link between S phase and mitosis. Proc Natl Acad Sci U S A *97*, 2579-2584.

Curran, S., Dey, G., Rees, P., and Nurse, P. (2022). A quantitative and spatial analysis of cell cycle regulators during the fission yeast cycle. Proc Natl Acad Sci U S A *119*, e2206172119.

Ermentrout, B. (2002). Simulating, Analyzing, and Animating Dynamical Systems: A Guide to XPPAUT for Researchers and Students. Society for Industrial and Applied Mathematics: Philadelphia.

Gillespie, D.T. (2007). Stochastic simulation of chemical kinetics. Annu Rev Phys Chem *58*, 35-55.

Keifenheim, D., Sun, X.M., D'Souza, E., Ohira, M.J., Magner, M., Mayhew, M.B., Marguerat, S., and Rhind, N. (2017). Size-Dependent Expression of the Mitotic Activator Cdc25 Suggests a Mechanism of Size Control in Fission Yeast. Curr Biol *27*, 1491-1497 e1494.

Mitchison, J.M. (2003). Growth during the cell cycle. Int Rev Cytol *226*, 165-258.

Ng, S.S., Anderson, M., White, S., and McInerny, C.J. (2001). mik1(+) G1-S transcription regulates mitotic entry in fission yeast. FEBS Lett *503*, 131-134.

Patterson, J.O., Basu, S., Rees, P., and Nurse, P. (2021). CDK control pathways integrate cell size and ploidy information to control cell division. Elife *10*.

Pedraza, J.M., and Paulsson, J. (2008). Effects of molecular memory and bursting on fluctuations in gene expression. Science *319*, 339-343.

Rudner, A.D., Hardwick, K.G., and Murray, A.W. (2000). Cdc28 activates exit from mitosis in budding yeast. J Cell Biol *149*, 1361-1376.

Shahrezaei, V., Ollivier, J.F., and Swain, P.S. (2008). Colored extrinsic fluctuations and stochastic gene expression. Mol Syst Biol *4*, 196.

Shahrezaei, V., and Swain, P.S. (2008). The stochastic nature of biochemical networks. Curr Opin Biotechnol *19*, 369-374.

Supplementary figures

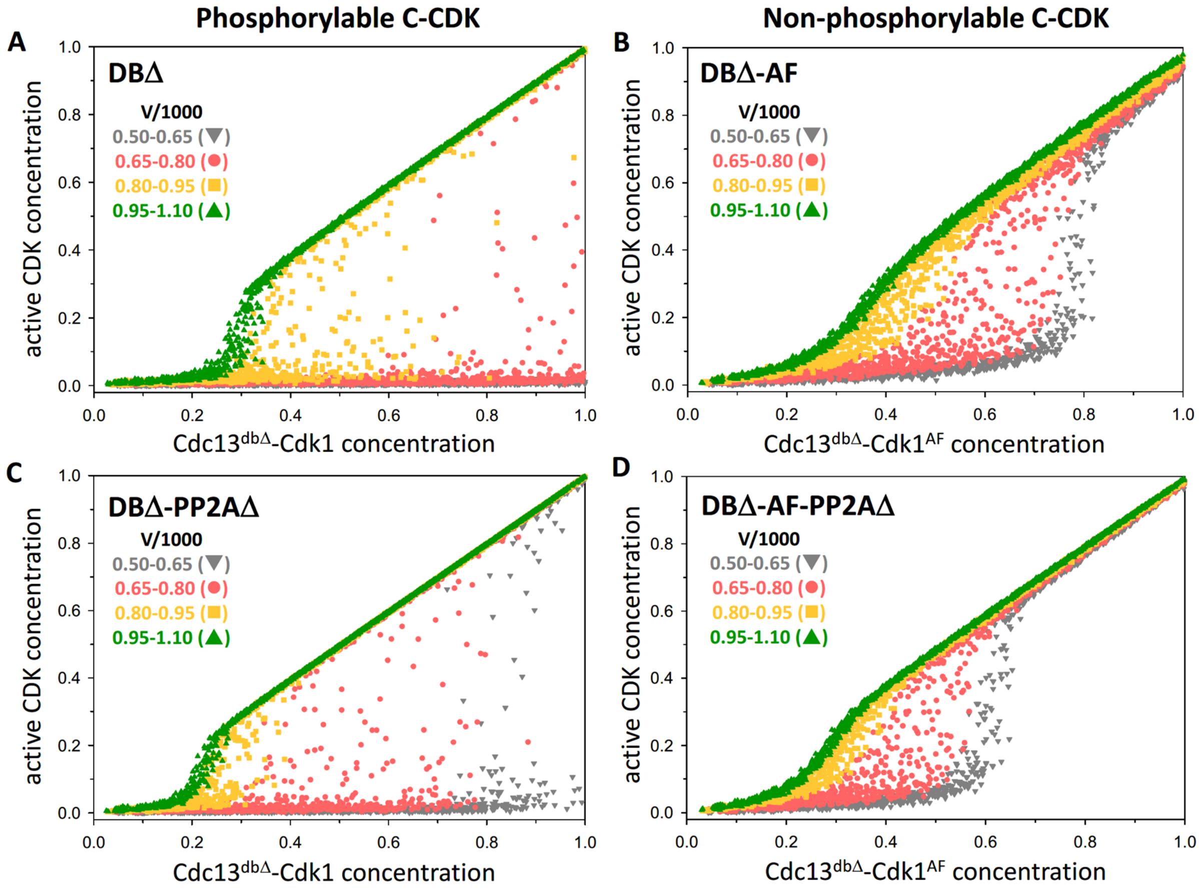

Figure S1: Stochastic simulations of the bistable mitotic-switch model. The concentration of the active form of CDK is plotted as a function of total fusion-protein concentration after induction of Cdc13$^{db\Delta}$-Cdk1 (left column: A & C) and Cdc13$^{db\Delta}$-Cdk1^{AF} (right column: B & D) in *pp2a*$^{+}$ (top row: A & B) and *pp2a*Δ-deleted background (bottom row: C & D).

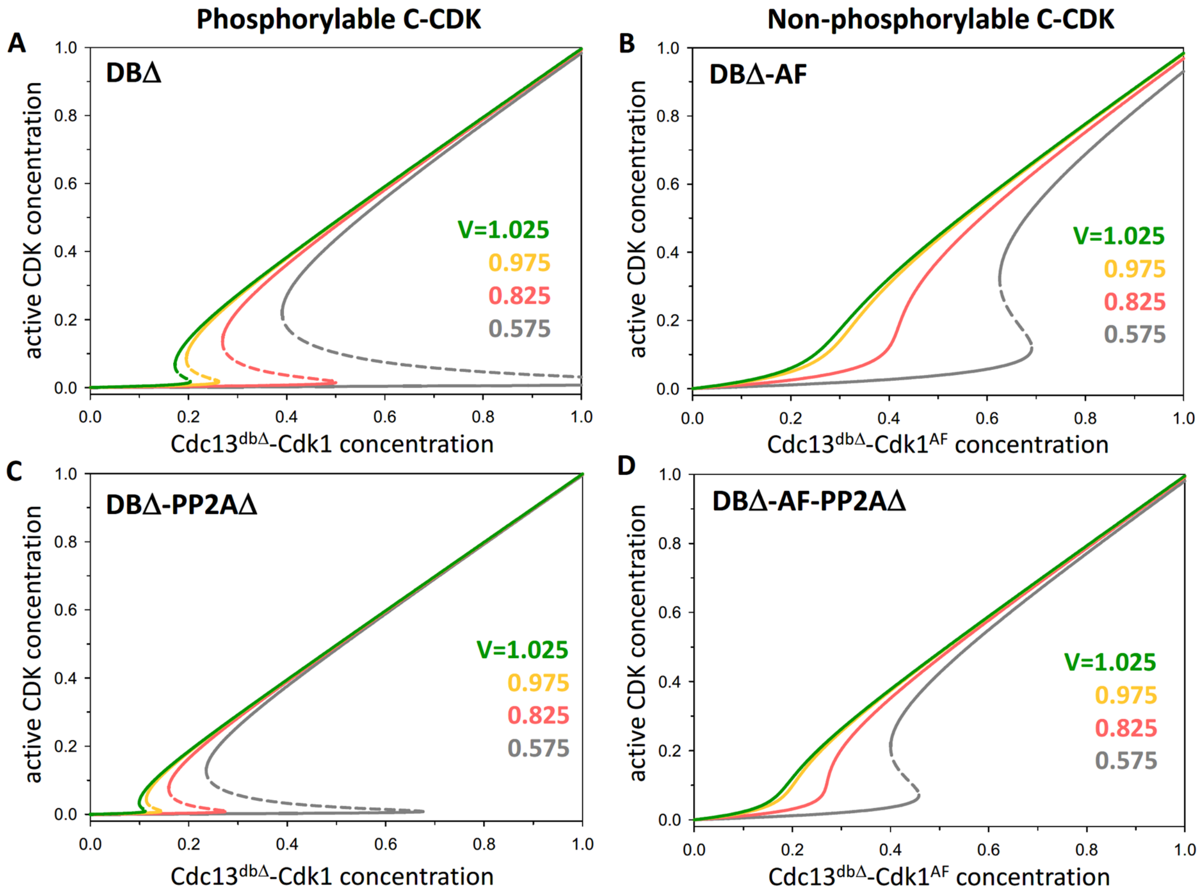

Figure S2: Dose-response curve for dependence of active CDK on total fusion-protein level. The concentration of the active form of CDK is plotted as a function of the total concentration of fusion protein after induction of Cdc13$^{db\Delta}$-Cdk1 (left column: A & C) and Cdc13$^{db\Delta}$-Cdk1^{AF} (right column: B & D) in *pp2a*$^{+}$ (top row: A & B) and *pp2a*Δ-deleted background (bottom row: C & D).

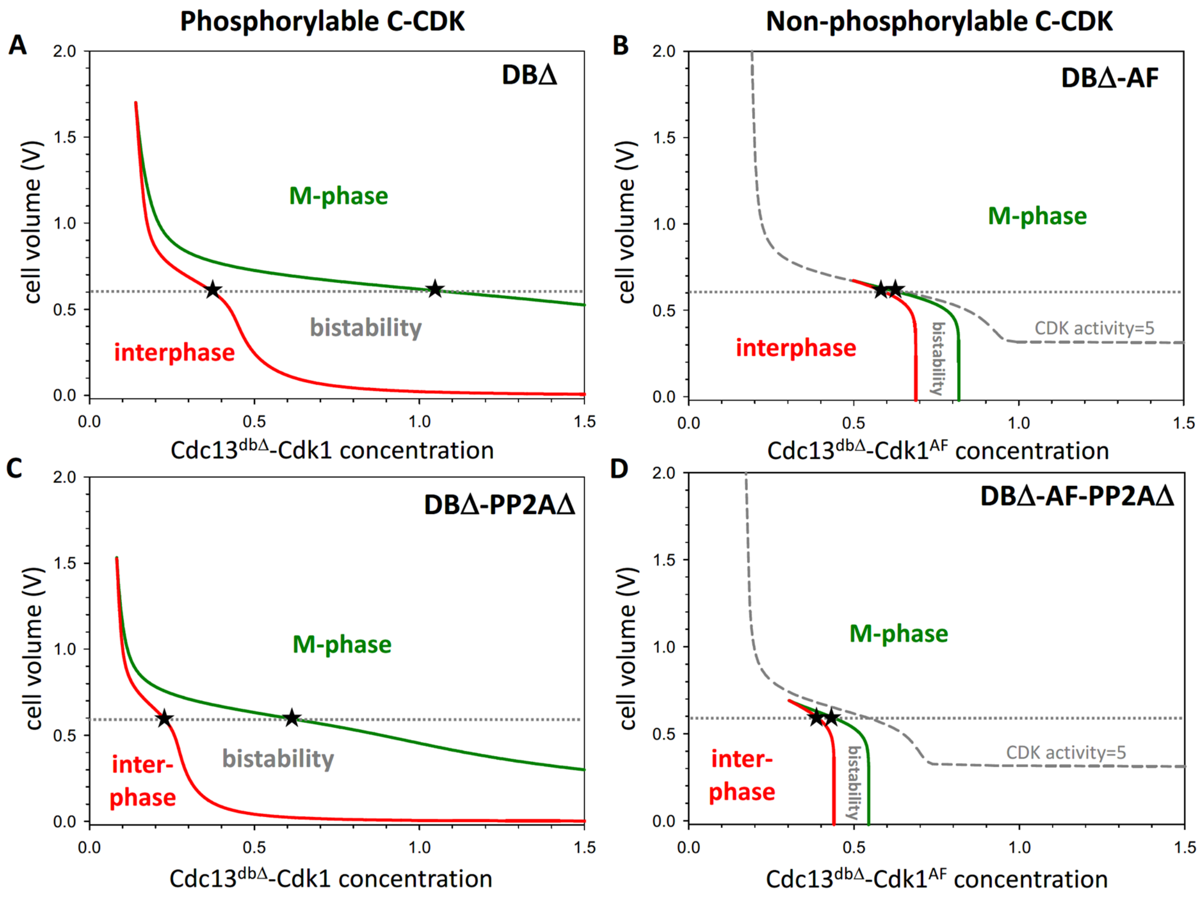

Figure S3: Two-parameter bifurcation diagrams (for the deterministic model) depicting the cell-size dependence of fusion-protein thresholds for mitotic entry (green curves) and exit (red curves. Induction of Cdc13$^{db\Delta}$-Cdk1 (left column: A & C) and Cdc13$^{db\Delta}$-Cdk1^{AF} (right column: B & D) in *pp2a*$^{+}$ (top row: A & B) and *pp2aΔ*-deleted background (bottom row: C & D). The horizontal lines at V=0.6 indicate the width of the bistable domain. The grey dashed lines in panels B and D indicate CDK activity =5 in the Cdk1^{AF} strains.

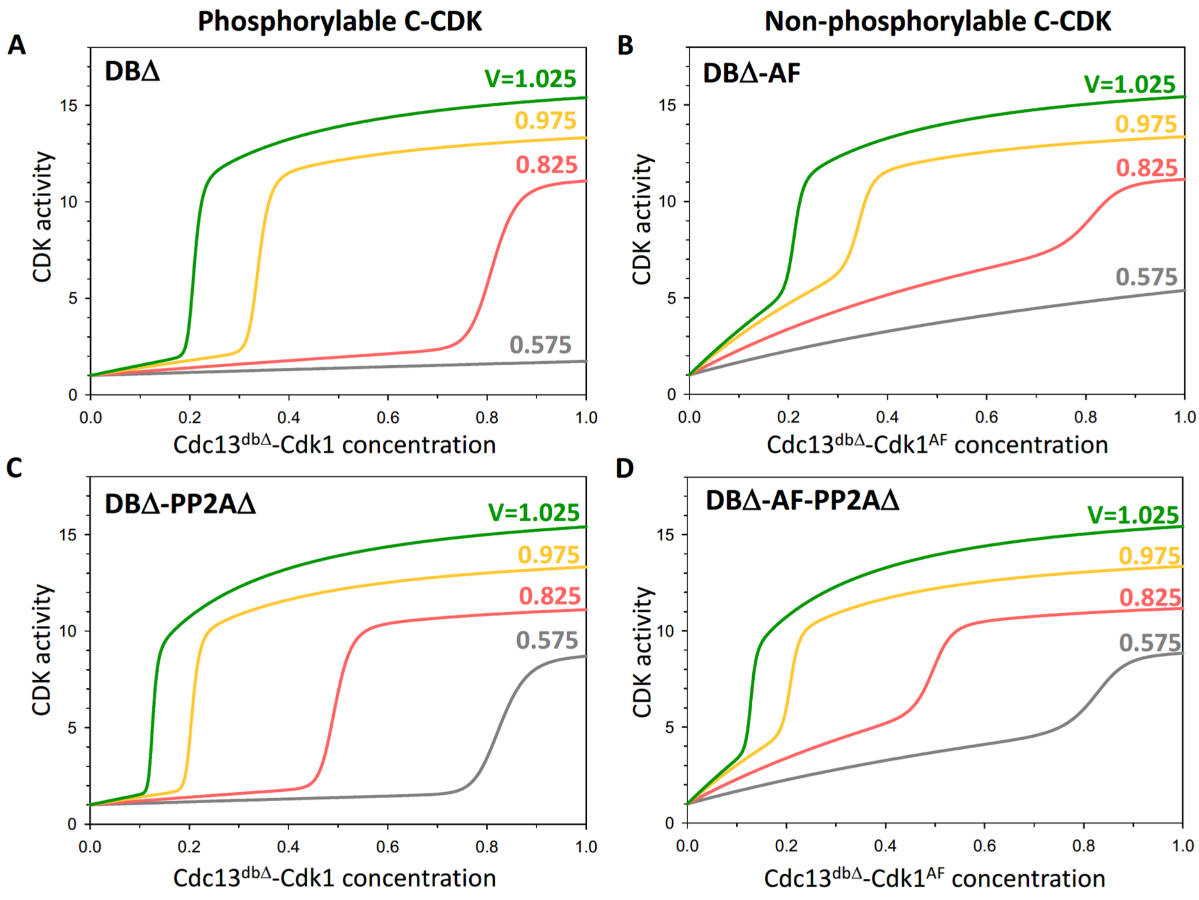

Figure S4: Dose-response curves for the dependence of CDK-activity sensor on fusion-protein level predicted by the reversible mitotic-switch model. CDK-activity sensor is plotted as a function of fusion-protein concentration after induction of Cdc13$^{db\Delta}$-Cdk1 (left column: A & C) and Cdc13$^{db\Delta}$-Cdk1^{AF} (right column: B & D) in *pp2a*$^{+}$ (top row: A & B) and *pp2aΔ*-deleted background (bottom row: C & D). CDK activities are calculated for cell size values in the middle of the cell-size bins.

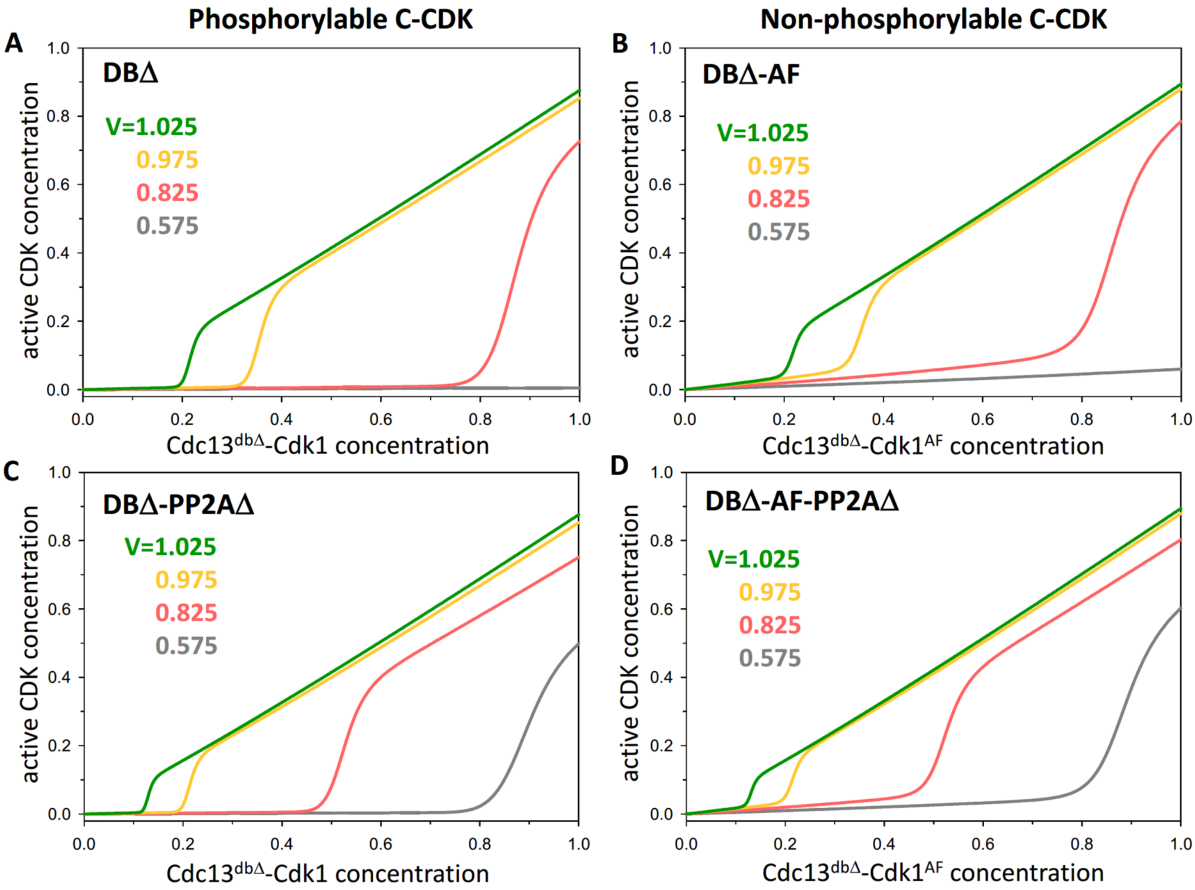

Figure S5: Dose-response curve for dependence of active CDK level on fusion-protein level in the reversible mitotic-switch model. The concentration of the active form of CDK is plotted as a function of the total concentration of fusion protein after induction of $Cdc13^{db\Delta}$-Cdk1 (left column: A & C) and $Cdc13^{db\Delta}$-$Cdk1^{AF}$ (right column: B & D) in *pp2a*$^{+}$ (top row: A & B) and *pp2aΔ*-deleted background (bottom row: C & D). CDK levels are calculated for cell size values in the middle of cell-size bins.

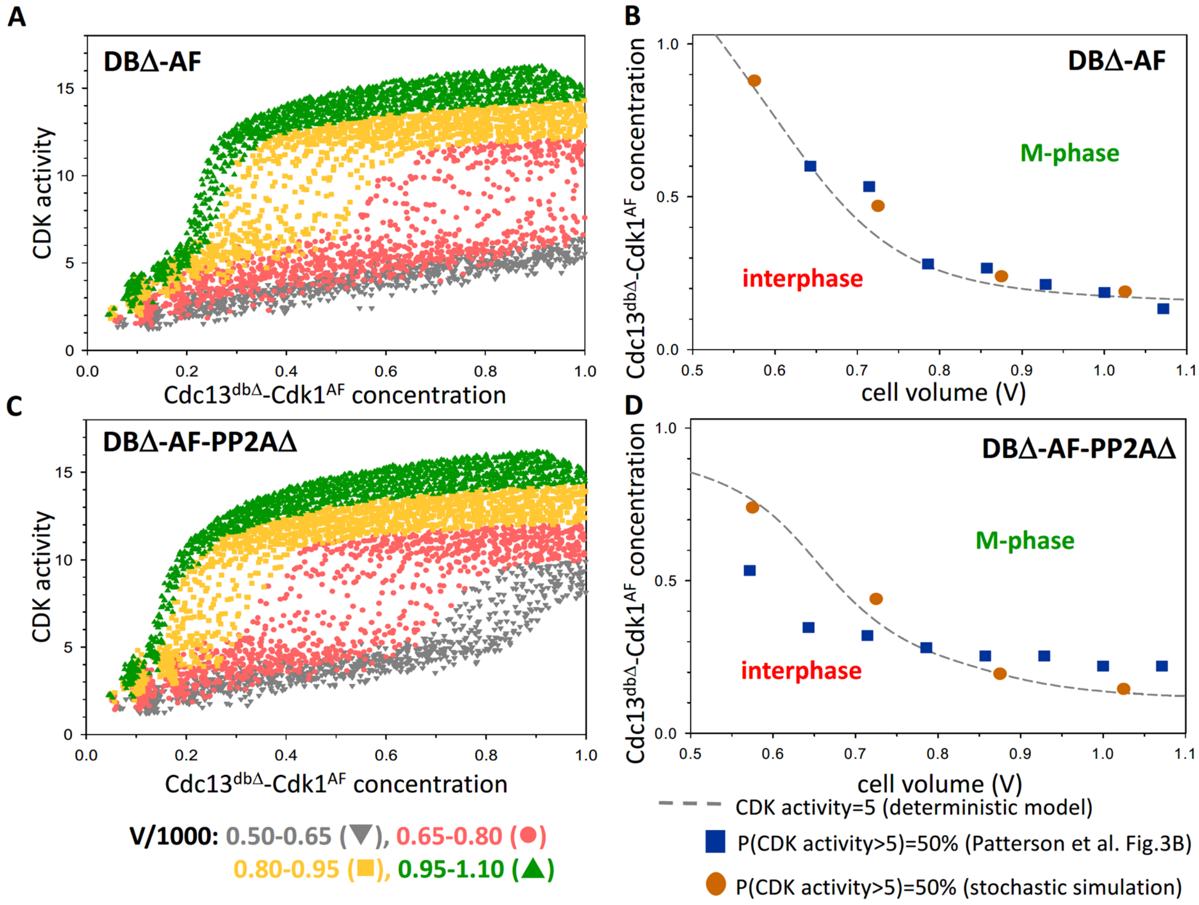

Figure S6: The reversible mitotic-switch model. (A, C) Stochastic simulations of Cdc13$^{db\Delta}$-Cdk1^{AF} induction in *pp2a*$^{+}$ and *pp2a*Δ-deleted backgrounds, respectively. (B, D) Cell size dependence of fusion-protein threshold of mitotic entry in *pp2a*$^{+}$ and *pp2a*Δ-deleted backgrounds, respectively. The dashed grey lines depict where CDK activity =5 in the deterministic model. The orange circles and blue squares indicate the fusion-protein levels where more than 50% of cells have CDK activity >5 in our stochastic simulations and in Patterson et al. (2021) experiments, respectively.

XPPAut models for fission yeast mitotic switch

Model simulations were performed using the freely available software XPP/AUTO (http://www.math.pitt.edu/~bard/xpp/xpp.html). The models are provided below in the form of .ode files readable by XPP. To use, copy the code into a text editor and save as [filename].ode.

```
# XPPAut model for fission yeast mitotic switch

# Differential equations
CDK' = Rcdc25*pCDK - kasswee1*CDK*Wee1 + (kdiswee1 + Riwee1 + kdwee1)*CDKWee1
pCDK' = kwee1*CDKWee1 - Rcdc25*pCDK
Wee1tot' = Rswee1 - kdwee1*Wee1tot
Wee1' = Rswee1 - kasswee1*CDK*Wee1 + (kdiswee1 + kwee1)*CDKWee1 - (Riwee1 +
kdwee1)*Wee1 + Rawee1*Wee1P
Wee1P2' = Riwee1*Wee1P - (Rawee1 + kdwee1)*Wee1P2
Cdc25tot' = kscdc25*V - kdcdc25*Cdc25tot
Cdc25' = kscdc25*V + Ricdc25*Cdc25P - (Racdc25 + kdcdc25)*Cdc25
Cdc25P2' = Racdc25*Cdc25P - (Ricdc25 + kdcdc25)*Cdc25P2
sensor' = eps*(V*kout/kout'*(kout'+kpsensor*AF*CDK)/(V*kout+kpsensor*AF*CDK) - sensor)

# Algebraic equations
CDKWee1 = CycBtot - CDK - pCDK
Wee1P = Wee1tot - CDKWee1 - Wee1 - Wee1P2
Cdc25P = Cdc25tot-Cdc25-Cdc25P2
Rswee1 = kswee1' + kswee1*Jwee1^n/(Jwee1^n + V^n)
Riwee1 = kiwee1*AF*CDK
Rawee1 = kawee1*PP2A
Racdc25 = kacdc25*AF*CDK
Ricdc25 = kicdc25*PP2A
Rcdc25  = kcdc25'*Cdc25tot + (kcdc25"-kcdc25')*Cdc25P2

p CycBtot=3, V=1, AF=1, PP2A=1
p kasswee1=5, kdiswee1=1, kwee1=1
p kswee1'=0.005, kswee1=0.02, Jwee1=0.65, n=10, kdwee1=0.005
p kiwee1=10, kawee1=1, kicdc25=1, kacdc25=10
p kscdc25=0.05, kdcdc25=0.05, kcdc25'=0.1, kcdc25"=5
p kout=1, kout'=0.06, kpsensor=10, eps=10

@ total=500,dt=0.5,meth=STIFF,xp=time,yp=sensor,xlo=0,xhi=500,ylo=0,yhi=1
@ NTST=150,NMAX=100000000,NPR=100000,DS=-0.001,BOUNDS=2000
@ DSMAX=0.01,DSMIN=0.001,PARMIN=0,PARMAX=3
@ AUTOXMIN=0,AUTOXMAX=1,AUTOYMIN=0,AUTOYMAX=20,AUTOVAR=sensor
done
```

```
# Gillespie's SSA for fission yeast mitotic switch

# Parameter values
p Vstart=500, V0=500, kscycb=0.02, kdcycb=0.002
p mu=0.005, AF=1, PP2A=1
p kasswee1=5, kdiswee1=1, kwee1=1
p kswee1'=0.005, kswee1=0.02, Jwee1=0.65, n=10, kdwee1=0.005
p kiwee1=10, kawee1=1, kicdc25=1, kacdc25=10
p kscdc25=0.05, kdcdc25=0.05, kcdc25'=0.1, kcdc25"=5
p kout=1, kout'=0.06, kpsensor=10, eps=10

init CDK=0, CDKWee1=0, Wee1P=0, Wee1P2=0, Cdc25P=0, Cdc25P2=0, CycBtot=1, Finish=1

Riwee1 = kiwee1/V*AF*CDK
Rawee1 = kawee1*PP2A
Ricdc25 = kicdc25*PP2A
Racdc25 = kacdc25/V*AF*CDK
Rcdc25 = kcdc25'*Cdc25tot/V + (kcdc25"-kcdc25')/V*Cdc25P2

V = Vstart*exp(mu*tr)
#  compute the cumulative reactions
# CycB synthesis & degradation
p1 = kscycb*V
p2  = p1 + kdcycb*CDK
p3  = p2 + kdcycb*CDKWee1
p4  = p3 + kdcycb*pCDK
# CDK activation & inactivation
p5  = p4+ kasswee1*CDK/V*Wee1
p6  = p5 + kdiswee1*CDKWee1
p7  = p6 + Riwee1*CDKWee1
p8  = p7 + kdwee1*CDKWee1
# pCDK activation & inactivation
p9 = p8 + kwee1*CDKWee1
p10 = p9 + Rcdc25*pCDK
# Wee1
p11 = p10 + kswee1'*V + kswee1*V*Jwee1^n/(Jwee1^n + (V/(2*V0))^n)
p12 = p11 + Rawee1*Wee1P
p13 = p12 + Riwee1*Wee1
p14 = p13 + kdwee1*Wee1
# Wee1P
p15 = p14 + kdwee1*Wee1P
# Wee1P2
p16 = p15 + Riwee1*Wee1P
p17 = p16 + Rawee1*Wee1P2
p18 = p17 + kdwee1*Wee1P2
# Cdc25
p19 = p18 + (kscdc25*V^2)/(2*V0)
p20 = p19 + Ricdc25*Cdc25P
p21 = p20 + Racdc25*Cdc25
p22 = p21 + kdcdc25*Cdc25
# Cdc25P
p23 = p22 + kdcdc25*Cdc25P
# Cdc25P2
p24 = p23 + Racdc25*Cdc25P
p25 = p24 + Ricdc25*Cdc25P2
p26 = p25 + kdcdc25*Cdc25P2

Cdc25tot=Cdc25+Cdc25P+Cdc25P2
Wee1tot=Wee1+CDKWee1+Wee1P+Wee1P2

# choose random #
s2=ran(1)*p26
z[1]=(s2<p1)
```

```
z[2..25]=(s2<p[j])&(s2>=p[j-1])
z[26]=(s2>p25)

# time for next reaction
tr'=tr-log(ran(1))/p26
CycBtot'=max(1,CycBtot+z1-z2-z3-z4)
CDK'=max(0,CDK+z1+z10-z2-z5+z6+z7+z8)
CDKWee1' =max(0,CDKWee1+z5-z6-z7-z8-z3-z9)
pCDK'=max(0,pCDK+z9-z10-z4)
Wee1'=max(0,Wee1+z11-z5+z6+z3+z9+z12-z13-z14)
Wee1P'=max(0,Wee1P+z7+z13-z12-z15-z16+z17)
Wee1P2'=max(0,Wee1P2+z16-z17-z18)
Cdc25'=max(0,Cdc25+z19+z20-z21-z22)
Cdc25P'=max(0,Cdc25P+z21-z20-z23-z24+z25)
Cdc25P2'=max(0,Cdc25P2+z24-z25-z26)
Finish'=max(0,Finish-z1+z2+z3+z4)

global 0 t
{Cdc25=kscdc25/2/V0/kdcdc25*Vstart^2;Wee1=(kswee1'+kswee1*Jwee1^n/(Jwee1^n+(Vstart/2/V0)^n))/kdwee1*Vstart}

# Auxiliary variables
aux sensor = V/(2*V0)*kout/kout'*(kout'+kpsensor*AF*CDK/V)/(V/(2*V0)*kout+kpsensor*AF*CDK/V)
aux V = Vstart*exp(mu*tr)
aux Cdc25tot=Cdc25+Cdc25P+Cdc25P2
aux Wee1tot=Wee1+CDKWee1+Wee1P+Wee1P2
aux CycBconc = CycBtot/V

# XPP instructions
@ bound=100000000,meth=discrete,total=1000000,njmp=1000
@ xlo=0,ylo=0,xhi=1,yhi=20
@ xp=CycBconc,yp=sensor
done
```
